# The evolution of the COVID-19 pandemic through the lens of google searches

**DOI:** 10.1038/s41598-023-41675-4

**Published:** 2023-11-13

**Authors:** Robert Marty, Manuel Ramos-Maqueda, Nausheen Khan, Arndt Reichert

**Affiliations:** 1 World Bank, Washington, DC, USA; 2 University of Hannover, Hanover, Germany

**Keywords:** Public health, Epidemiology

## Abstract

Real-time data is essential for policymakers to adapt to a rapidly evolving situation like the COVID-19 pandemic. Using data from 221 countries and territories, we demonstrate the capacity of Google search data to anticipate reported COVID-19 cases and understand how containment policies are associated with changes in socioeconomic indicators. First, search interest in COVID-specific symptoms such as “loss of smell” strongly correlated with cases initially, but the association diminished as COVID-19 evolved; general terms such as “COVID symptoms” remained strongly associated with cases. Moreover, trends in search interest preceded trends in reported cases, particularly in the first year of the pandemic. Second, countries with more restrictive containment policies experienced greater search interest in unemployment and mental health terms after policies were implemented, indicating socio-economic externalities. Higher-income countries experienced a larger increase in searches related to unemployment and a larger reduction in relationship and family planning keywords relative to lower-income countries. The results demonstrate that real-time search interest can be a valuable tool to inform policies across multiple stages of the pandemic.

## Introduction

The COVID-19 pandemic and the associated policy responses have rapidly evolved. Across the world, people have had to adapt in multiple ways. Whether it was adopting preventative measures such as masks or testing; understanding symptoms, risks, and treatment options; adapting lifestyles to social distancing measures; or dealing with business closures and unemployment, people throughout the world have been continuously learning how to adapt to the pandemic. One of the first places people turn to learn about coronavirus symptoms, new policies, and vaccines are online Google searches. Google holds 92% of the search engine market share^1^, and in 2020, “coronavirus” was the most commonly searched term in Google^2^. These searches generate a vast amount of publicly available information about people’s symptoms, fears, concerns, and demand for information. These real-time data can have high value for policymakers who aim to better understand, monitor, and react to citizens’ experiences. Google search patterns can provide crucial information during times when other sources, such as traditional household surveys, are too costly or not feasible due to infection risks. Additionally, Google search data is always up-to-date, making it particularly useful for real-time decision-making^3^.

Previous research has shown the potential of Google search interest to inform different stages of the pandemic, including anticipating rising COVID-19 cases or understanding potential consequences of containment policies^4–9^. However, these studies have focused on either a single or a limited set of countries, limiting their capacity to understand variation across countries and their characteristics—such as by income level or a country’s policy response to COVID-19. Leveraging methodologies from existing research, this paper extends the analysis to the global level. We extract and analyze data across all countries with available data—encompassing 221 countries and territories throughout the world (henceforth, we use “countries” to refer to both countries and territories). Doing so allows us to examine heterogeneity of the use of Google search interest data across countries. Specifically, we examine the capacity to use this real-time, big data source for two objectives: (1) anticipate rising COVID-19 cases, and (2) understand how containment policies are associated with select social and economic indicators. In our analysis, we examine the extent to which results vary across countries depending on their characteristics, such as income level and geographic region.

To make the analysis possible worldwide, we develop a method to identify the most popular language for Google searches in each country, and automatically translate search terms into each country’s most popular language using the Google Translate API. We then systematically query the search terms from Google Trends for each country and analyze these data to generate decision-focused evidence that responds to the situation on the ground and can contribute to informing countries’ and citizens’ responses to the pandemic. We query data using the Google Trends web-based platform, facilitated by the gtrendsR R package^10^. Some researchers instead rely on the Google Health Trends API, which has advantages in facilitating comparisons between regions across many keywords^11,12^; however, this data source is not easily accessible for a public audience, which may pose difficulties in its use in a policy context. Instead, we rely on data from the web-based platform to facilitate replicating the analysis. We also implement methodologies to overcome limitations to making cross-country comparisons from the web-based platform (as detailed in the methods section). Our analysis thus relies on the same data that researchers and practitioners alike can easily and quickly search through the Google Trends website.

As to the first objective, we find that search interest in COVID-19-specific symptoms such as “loss of smell” and “loss of taste”, as well as select more general searches such as “COVID symptoms”, strongly correlate with and precede reported coronavirus cases across countries. These results build off of findings from studies focusing on a limited set of countries or regions, both in higher income^13,14^ and lower income countries^4–8,15^. By systematically analyzing all countries with available data, our results demonstrate that the estimated correlations are as applicable to lower-income countries as they are to higher-income countries.

Prior work on other diseases has also emphasized the importance of checking and recalibrating models when forecasting illness, particularly as the nature of illnesses changes^16,17^—such as with new variants of COVID-19. To this point, we separately examine 2020, 2021, and 2022 data. The association between COVID-19 cases and search interest in “loss of smell” and “loss of taste” diminished over time, particularly in 2022 as Omicron became the dominant strain and loss of smell and taste became less common symptoms^18^. More general terms such as “COVID symptoms” remained strongly associated with reported cases throughout the 3 years.

To examine the second research question, we implement a difference-in-differences approach that compares trends in search interest before and after the date containment policies were implemented in both the year containment policies were implemented and in the year before. Our results show that containment policies are significantly associated with greater interest in search terms including unemployment, mental health, and social distancing, and lower interest in search terms for personal relationships and family planning. These results are consistent with studies that focus on analyzing COVID-19 policies within the United States and Western Europe^19–26^.

By leveraging data across all countries with available data, we examine how the association of COVID-19 policies with select indicators varies by country characteristics and the type of policies implemented. We find that containment policies were associated with larger increases in select unemployment and unemployment resource keywords (e.g., “unemployment office”) in higher-income countries relative to lower-income countries. In addition, higher-income countries saw a larger reduction in search interest in most relationship and family planning keywords after containment policies were implemented. Furthermore, our results suggest that countries with more restrictive containment policies experienced more pronounced mental health and social consequences (i.e., greater search interest in select mental health keywords and less interest in select relationship and family planning keywords).

Our findings demonstrate that publicly available, real-time data from Google searches are a useful tool for monitoring the spread and consequences of the COVID-19 pandemic across the entire world. These data are immediately available for free across a majority of countries even in cases where access to the internet is less widespread. In some cases, the data may also be used as a contrast to administrative data, such as in instances where countries misreport or altogether deny the presence of COVID-19 cases.

## Results

### Google search interest correlating with and preceding COVID-19 cases

**Figure 1:**
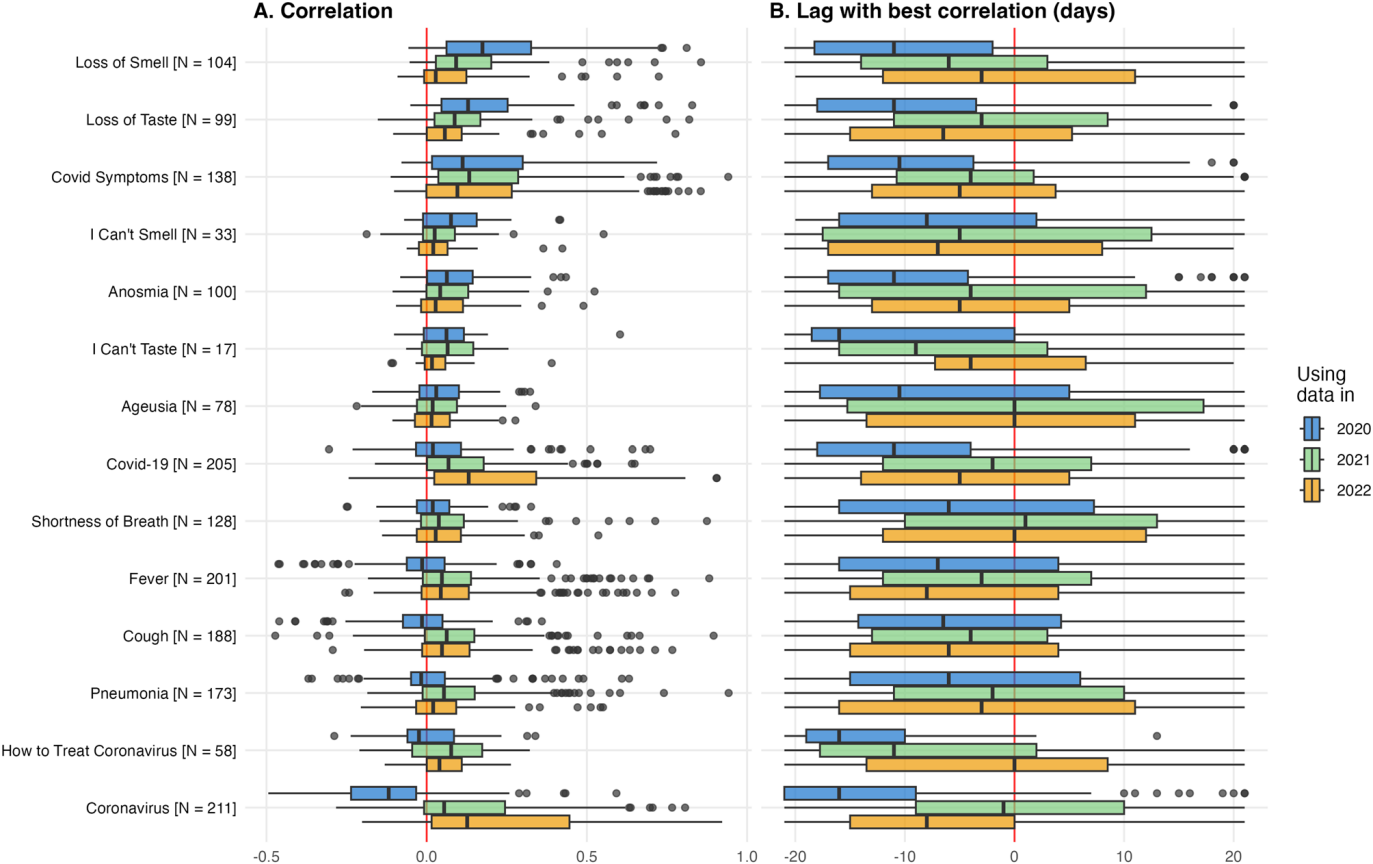
Search interest correlating with and anticipating COVID-19 cases. Panel (**A**) shows the correlation between search interest and reported COVID-19 cases. Panel (**B**) shows the lead/lad value of COVID-19 cases that produced the highest correlation with search interest. ‘N’ indicates the number of countries with available data. The boxplots include: center line, median; box limits, upper and lower quartiles; whiskers, $\times$1.5 interquartile range; points beyond whiskers, outliers.

**Figure 2:**
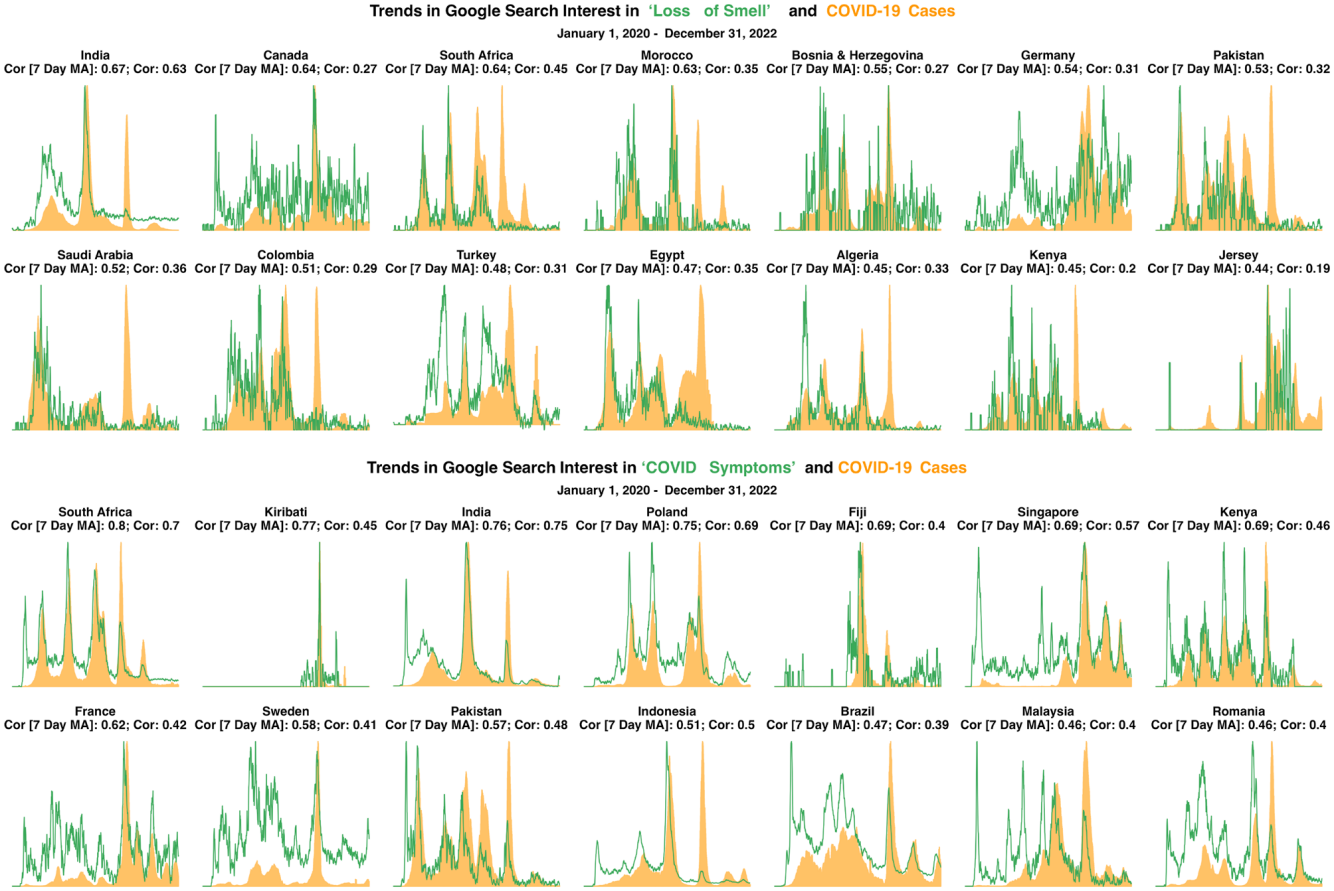
Trends between reported COVID-19 cases and search interest in (1) “Loss of Smell” (top) and (2) “COVID Symptoms” (bottom) for countries with the top correlations between cases and search interest. To more clearly show trends, the 7-day moving averages of search interest and cases are shown.

**Table 1:** Explaining correlation between search interest in loss of smell and COVID-19 cases, using data from 2020 and 2021.

	*Dependent variable:*							
	Correlation							
	(1)	(2)	(3)	(4)	(5)	(6)	(7)	(8)
Total COVID-19 cases, log	$$0.02^{***}$$ (0.01)						$$0.03^{***}$$ (0.01)	$$0.03^{***}$$ (0.01)
Per pop. using internet		0.0000 (0.001)						0.0002 (0.002)
Mobile cell sub. per 100			0.0000 (0.0005)					0.0002 (0.001)
GDP per cap, log				− 0.004 (0.01)			− 0.04 (0.03)	− 0.04 (0.04)
Low income					− 0.01 (0.06)		− 0.03 (0.13)	0.03 (0.13)
Lower middle income					0.05 (0.04)		0.04 (0.09)	0.04 (0.09)
Upper middle income					0.005 (0.04)		0.01 (0.06)	0.01 (0.06)
Europe and Central Asia						− 0.03 (0.05)	− 0.01 (0.05)	− 0.01 (0.06)
Latin America and Caribbean						− 0.06 (0.06)	− 0.03 (0.06)	− 0.03 (0.06)
Middle East and North Africa						0.02 (0.06)	0.04 (0.06)	0.04 (0.06)
North America						$$0.33^{***}$$ (0.11)	$$0.34^{***}$$ (0.11)	$$0.35^{***}$$ (0.12)
South Asia						$$0.20^{**}$$ (0.10)	0.14 (0.10)	0.15 (0.10)
Sub-Saharan Africa						− 0.07 (0.06)	− 0.06 (0.06)	− 0.06 (0.07)
Constant	$$-0.18^{*}$$ (0.10)	$$0.13^{***}$$ (0.05)	$$0.13^{**}$$ (0.06)	0.17 (0.11)	$$0.12^{***}$$ (0.03)	$$0.15^{***}$$ (0.05)	0.05 (0.34)	0.05 (0.36)
Observations	112	106	110	107	109	112	107	105
Adjusted $\hbox {R}^{2}$	0.08	− 0.01	− 0.01	− 0.01	− 0.01	0.14	0.22	0.20

$$^{*}$$p < 0.1; $$^{**}$$p < 0.05; $$^{***}$$p < 0.01.

**Table 2:** Explaining the lead/lag value that produced the highest correlation between search interest in loss of smell and COVID-19 cases, using data from 2020 and 2021.

	*Dependent variable:*							
	Best lag							
	(1)	(2)	(3)	(4)	(5)	(6)	(7)	(8)
Total COVID-19 cases, log	0.07 (0.52)						− 0.33 (0.69)	− 0.44 (0.71)
Per Pop. using internet		0.03 (0.05)						0.06 (0.13)
Mobile cell sub. per 100			$0.06^{*}$ (0.03)					0.05 (0.05)
GDP per cap, log				0.56 (0.90)			0.19 (2.51)	− 1.13 (2.96)
Low income					− 4.99 (4.15)		− 14.11 (9.49)	$-12.75$ (9.74)
Lower middle income					− 1.36 (2.87)		− 5.06 (6.57)	− 4.74 (6.65)
Upper middle income					− 1.44 (2.80)		− 0.63 (4.33)	− 1.48 (4.45)
Europe and Central Asia						− 0.11 (3.91)	− 0.97 (4.08)	− 0.74 (4.20)
Latin America and Caribbean						$$-8.58^{**}$$ (4.07)	$$-9.92^{**}$$ (4.31)	$$-9.46^{**}$$ (4.38)
Middle East and North Africa						− 1.78 (4.27)	− 0.56 (4.37)	− 1.35 (4.63)
North America						3.00 (8.38)	− 1.26 (8.57)	3.43 (8.87)
South Asia						1.83 (7.12)	3.50 (7.28)	4.40 (7.54)
Sub-Saharan Africa						3.50 (4.13)	$8.26^{*}$ (4.84)	$8.57^{*}$ (5.08)
Constant	− 7.71 (7.15)	$$-8.21^{**}$$ (3.22)	$$-13.41^{***}$$ (3.99)	− 11.57 (8.01)	$$-5.41^{***}$$ (1.98)	− 5.50 (3.42)	- 0.32 (25.51)	3.58 (26.78)
Observations	112	106	110	107	109	112	107	105
Adjusted $\hbox {R}^{2}$	− 0.01	− 0.01	0.02	− 0.01	− 0.01	0.09	0.12	0.12

$$^{*}$$p < 0.1; $$^{**}$$p < 0.05; $$^{***}$$p < 0.01.

Search interest for symptoms more unique to COVID-19—particularly “loss of smell” and “loss of taste”—had the strongest correlation to reported COVID-19 cases (figure 1). However, the correlation diminished over time, particularly in 2022 as Omicron became the dominant variant and these symptoms became less common^18^. In 2020 and 2021, the 25th percentile of correlations using “loss of smell” and “loss of taste” across countries was above zero and the 75th percentile was near 0.2—with some countries seeing a correlation above 0.8. For search interest in loss of smell and taste, the 25th percentile and median correlation in 2021 were slightly lower than in 2020, but still above zero, indicating that high COVID-19 rates still generated large search interest in loss of smell and taste over a year into the pandemic—even as the general public may have become more knowledgeable about COVID-19 symptoms, such as loss of smell and taste, later in the pandemic. In 2022, the median correlation using loss of smell and taste drops to near 0. Appendix S4 compares the distribution of correlations using the raw correlations and the correlations using the optimal lag; correlations increase by about 0.1, but the overall patterns stay the same. As correlations at the annual level could mask more granular trends, Appendix S9 shows results using 6-month periods. Correlations using “loss of smell” and “loss of taste” steadily diminish over time, while correlations using “COVID symptoms” stay relatively consistent, until the correlation drops in the second half of 2022.

Low correlations between reported COVID-19 cases and Google search interest should not necessarily be interpreted as Google search interest serving as poor indicator of COVID-19 cases. Issues with reported cases could also lead to low correlations. COVID-19 cases have failed to be diagnosed or reported—with underreporting likely worse earlier in the pandemic^27,28^. Low testing capacity can lead to underreporting, but political motivations have also led to underreporting, such as governments wanting to demonstrate that COVID-19 was under control^29^. Consequently, high search interest in COVID-19 keywords but low reported cases could suggest issues with underreporting. An alternate measure of the burden of COVID-19 is excess mortality, which can help to overcome issues of reporting^27,30^. Excess mortality helps overcome issues in reported COVID-19 case data as the indicator does not rely on COVID-19 testing capacity. In addition, excess mortality helps overcome issues of reported COVID-19 death data as different countries both had different capacities to identify those who died from COVID-19, and countries had different standards for how a COVID-19 related death was identified^30,31^. We test whether our results are sensitive to using reported COVID-19 cases versus excess mortality, finding that results are similar (see Appendix S11). Google search interest remains strongly correlated with both reported COVID-19 cases and excess mortality, with search interest in search terms such as “loss of smell”, “loss of taste”, and “COVID-19 symptoms” having the strongest correlation with both COVID-19 cases and excess mortality. We rely on reported COVID-19 cases for our main analysis for two reasons: first, doing so follows similar studies that test the correlation of Google search interest with report COVID-19 cases^13,14,32–34^; and second, reported COVID-19 cases are available at a daily level (instead of monthly excess mortality data) and, as of this writing, are available for a longer time period; excess mortality data from the WHO goes through 2021, while our analysis of COVID-19 cases extends through 2022^35^.

As search interest in loss of smell and taste became less associated with reported cases over time, the correlation between “COVID symptoms” and reported cases remained strong throughout 2020 - 2022 (the 75th percentile correlation in 2020, 2021, and 2022 was 0.30, 0.29, and 0.27). Figure 2 shows trends in reported COVID-19 cases and “loss of smell” (top) and “COVID symptoms” (bottom) between January 1, 2020 and December 31, 2022 for the countries with the highest correlations (appendices S6 and S7 show trends for all countries with available data). The figure shows search interest for both search terms rising and falling with reported cases. In many countries, search interest in “loss of smell” stops moving with cases in 2022, while search interest in “COVID symptoms” continues to move with cases in many countries. For example, search interest in “loss of smell” moved closely with reported cases in the first three waves of COVID-19 in South Africa. However, Omicron was first reported in South Africa in November, 2021^36^; in waves of COVID-19 after November 2021, South Africa saw little search interest in “loss of smell”, but search interest in “COVID symptoms” continued to move with cases.

Search interest for “coronavirus” and “how to treat coronavirus” flip from being negatively correlated with cases in most countries in 2020 to positively correlated in most countries in 2021 and 2022. We hypothesize that global news may have been a larger factor in driving search interest in these general terms 2020, while searches in 2021 and 2022 may have been more driven by country-specific news and personal experiences about COVID-19. Bento et al.^19^ shows that early in the pandemic, initial case reports of COVID-19 led to higher search interest for terms like “coronavirus” across US states—indicating that local experiences contributed to search interest. However, Appendix S8 shows that search interest in “coronavirus” surged across nearly all countries as the pandemic began, suggesting that the initial media reports of the pandemic contributed to global search interest in general terms “coronavirus.”

Searches for more general symptoms—“fever” and “pneumonia”—see lower median and average correlation values compared to more COVID-19 specific searches; however, the distribution of correlations using “fever” and “pneumonia” are large. In some countries the correlation is close to one, indicating that search terms for more general symptoms can work well in specific contexts. Appendix S5 shows the spatial distribution of correlations in “loss of smell” and a more general symptom (“fever”), illustrating (1) how the correlation between COVID-19 cases and search interest in “loss of smell” is strong throughout different geographic regions and (2) the countries where search interest in “fever” correlates strongly with cases (e.g., India and Cuba).

Panel 1B shows the distribution in the lag/lead value of COVID-19 cases that had the best correlation with search interest. Across all search terms, the majority of countries with available data had a negative optimal lag—indicating that trends in search interest precede trends in reported COVID-19 cases. In addition, across search terms, the median lag value was more negative in 2020 than in 2021; in 2020, search terms anticipated COVID-19 cases by a larger number of days than in 2021. This result may indicate that, as the pandemic progressed, testing improved and countries were better able to monitor real-time caseloads. Using data in 2020, the median optimal lags for both loss of smell and loss of taste are − 11 days; using data since 2021, the values are about − 3 to − 6 (see Appendix S4).

Table 1 shows regressions results examining the type of countries where the correlation between reported cases and search interest in “loss of smell” is largest. Columns (1), (7), and (8) show that search interest is more correlated with reported cases in countries that saw a larger number of cases; a 1% increase in COVID-19 cases is associated with a 0.02–0.03 increase in the correlation coefficient (p < 0.01). The other variables we explore—such as internet usage, mobile subscriptions, GDP per capita, and income level—are not significantly associated with the correlation. North America (comprising just the US and Canada) and South Asia tend to see higher correlations than other geographic regions. Appendix S10 shows that results are largely consistent when using search interest in the terms with the second and third highest median correlations: “loss of taste” and “COVID symptoms”.

While the results point to total COVID-19 cases and geographic region explaining some of the variation in the correlation, the models explain—at best—22% of the variation in the correlation. Other factors could also explain variation in the association of Google Search interest to reported cases. For example, while the results show internet access is not associated with the correlation, bias in the type of people who access internet (e.g., by income level or by urban and rural locations) could impact the association between Google search data and reported cases. While Google holds 92% of the search engine market share globally^1^, variation in the popularity of Google across countries could also be important. Moreover, Google search interest not moving with reported cases could also result from issues with COVID-19 data, such as a country not capturing all cases due to limited diagnostic capabilities or—in the extreme case—not releasing case data, such as in Tanzania^37^.

Table 2 shows results when regressing indicators on the optimal lag of the correlation. The majority of country-level indicators are not significantly associated with the best lag. Across regions, Latin America and the Caribbean have, on average, a greater lag between Google search interest and reported COVID-19 cases than other regions.

### Association of containment policies with changes in economic, mental health, and social search interest indicators

**Figure 3:**
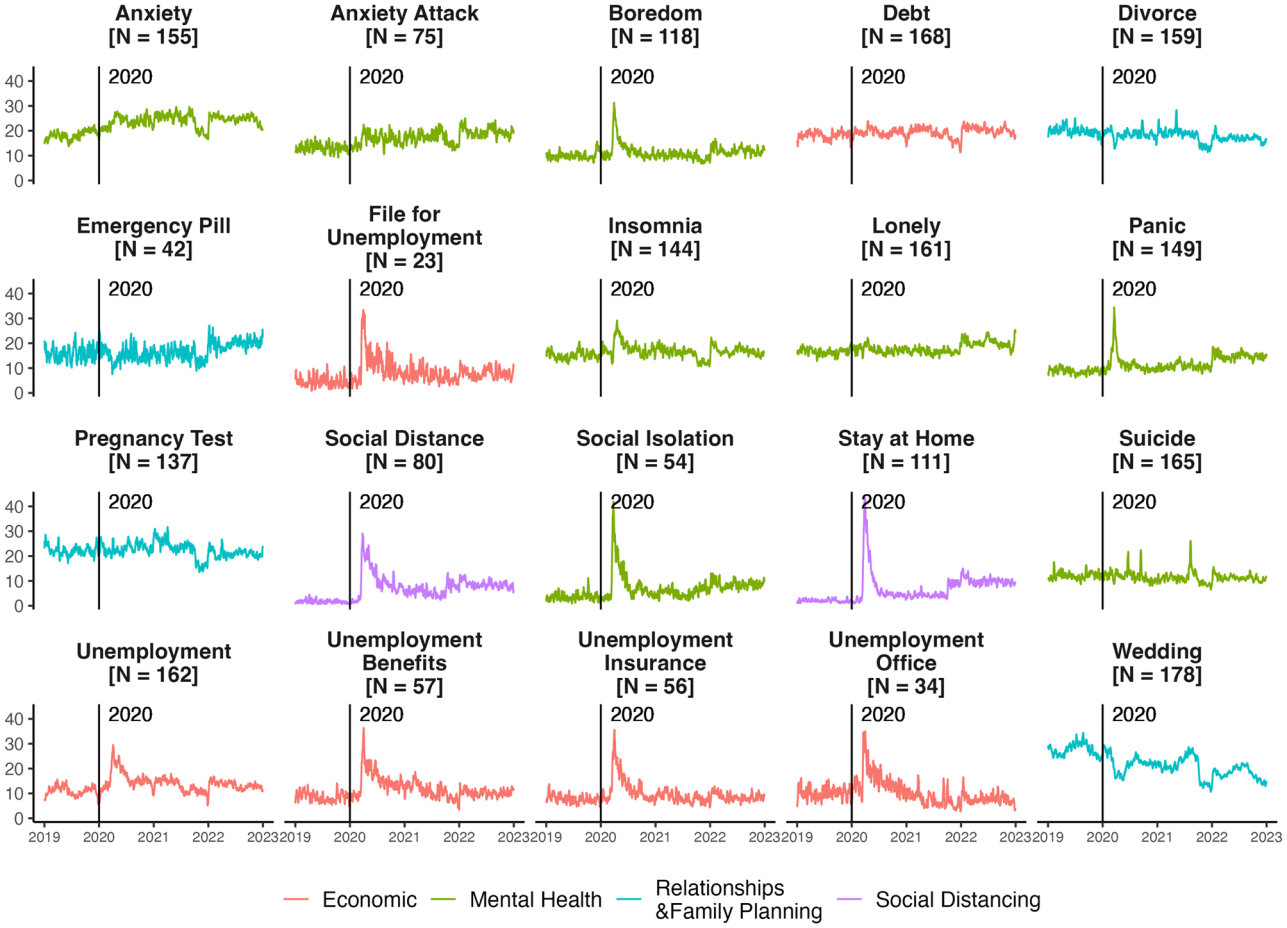
Trends in search interest in social, economic, and mental health keywords from 2019 to 2022. To show trends more clearly, the 7-day moving average of search interest is shown. ‘N’ indicates the number of countries with available data.

In this section, we evaluate three main questions. First, we implement a difference-in-differences study, accompanied by an event study, to understand the association of COVID-19 policies with changes in search interest across topics related to economic outcomes, mental health, social distancing, and relationships and family planning. Second, we explore heterogeneities in results across countries’ income levels. Third, we analyze the heterogeneous effect of containment policies depending on the level of economic support and restrictions to mobility across countries, also using a difference-in-difference design. Throughout the analysis, we rely on models that rely on data 90 days before and after the date of the first containment policy; Appendix S13 shows that these results are largely consistent when estimating models that rely on data 30, 60, 120 and 180 days before and after the date of the first containment policy. Using a 90-day (about 3 month) threshold follows from^23^, who examine trends in search interest in fertility and relationship search terms 3 months after lockdowns in the US and Europe. A 90-day threshold allows focusing the analysis on changes in outcomes after the first containment policies were implemented. However, we also report long-term trends in indicators (Fig. 3).

**Figure 4:**
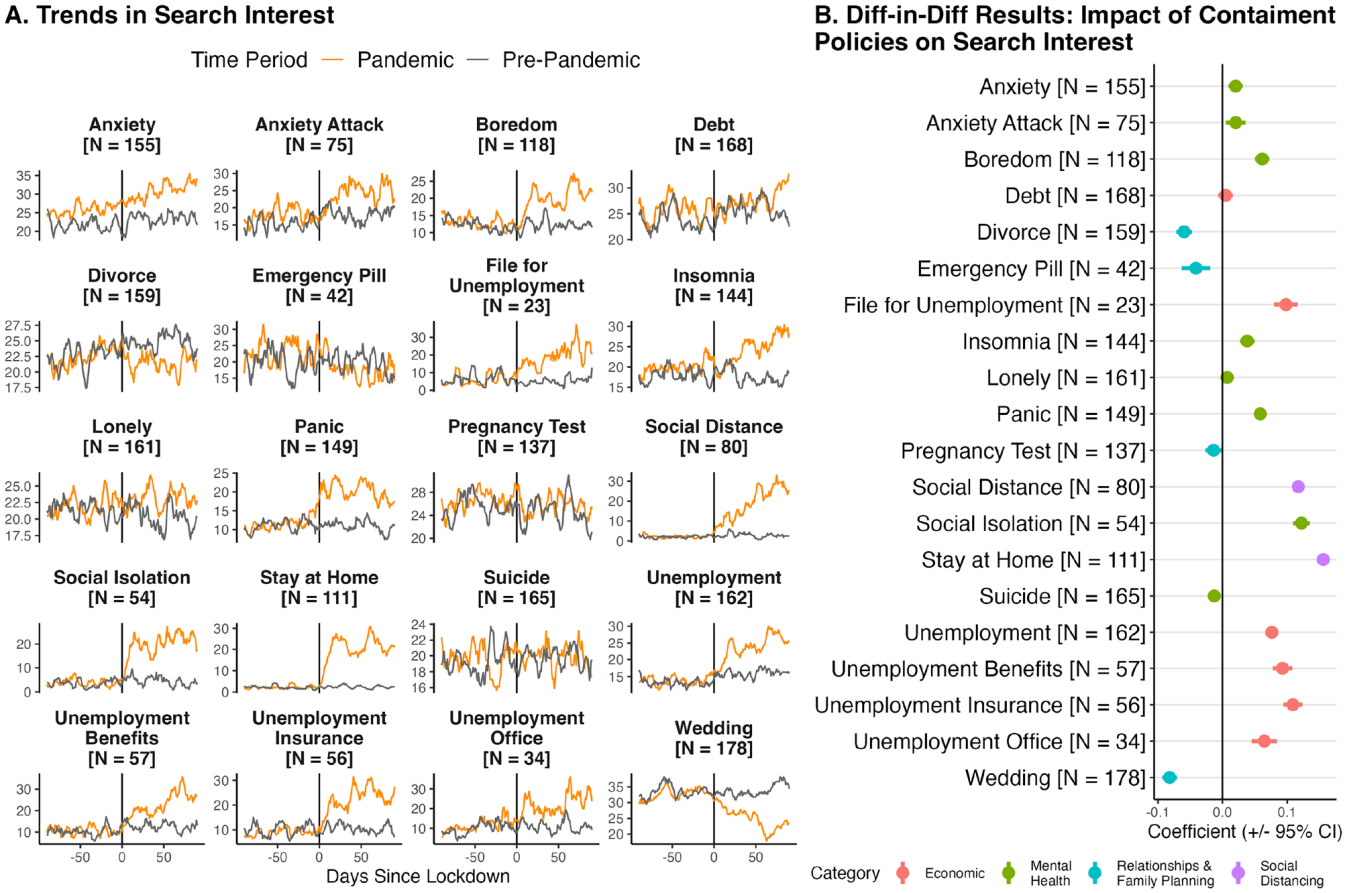
Association of COVID-19 policies and search interest: results pooling all countries. Point estimates and 95% confidence intervals are shown. To show trends more clearly, the 7-day moving average of search interest is shown in panel (**A**). ‘N’ indicates the number of countries with available data.

When considering all countries, we find that containment policies were associated with an increase in unemployment, mental health, and social distancing search terms and a decrease in relationship and family planning search terms (Fig. 4; Appendix S12 shows that these results are consistent when using an event study approach). These findings are in line with observed consequences of the pandemic, such as many countries experiencing high unemployment rates and worsening mental health conditions. For instance, Aknin et al.^39^ finds that higher policy stringency was associated with higher mean psychological distress scores and lower life evaluations across 15 countries. Using a sample of 46 countries, Morris et al.^40^ finds that containment measures have a strong impact on unemployment; increasing stringency levels to the median level results in unemployment increasing by 1.87 percentage points. A growing literature shows COVID-19 and lockdowns associated with lower psychological well-being—including higher anxiety and depression—among both health care workers and the general public^41^. Growth in search interest in select mental health terms (e.g., “panic” and “boredom”) returned to pre-pandemic levels relatively quickly (by mid-2020), while select economic indicator terms only return to pre-pandemic levels until 2021 or 2022 (Fig. 3).

**Figure 5:**
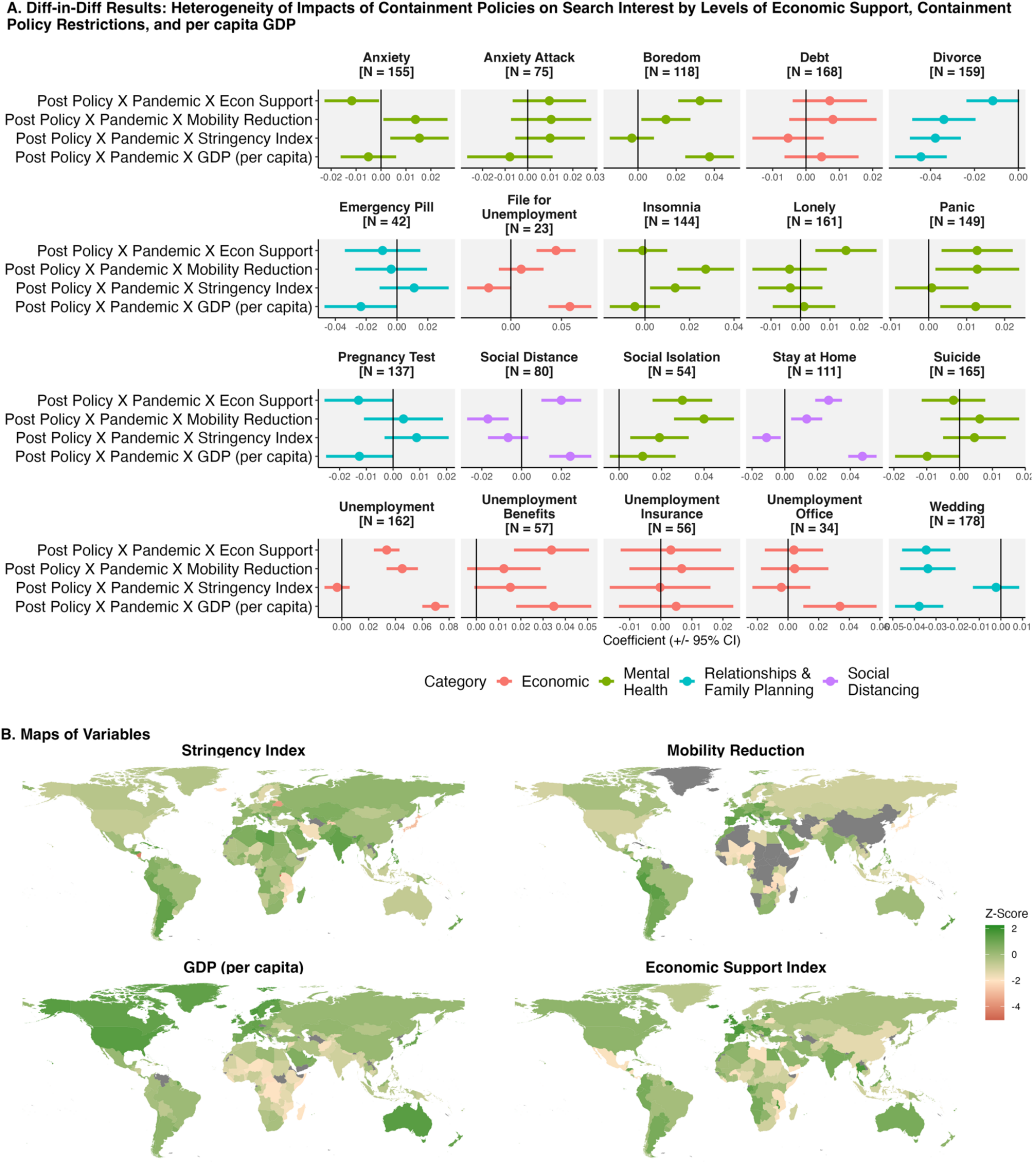
Association of COVID-19 policies with search interest: difference-in-differences results that explore heterogeneity of results across containment policy restrictiveness, economic support, and GDP per capita. Each coefficient comes from a separate regression. The stringency index comes from the University of Oxford COVID-19 Government Response tracker, a composite measure of the restrictiveness of policy measures. Mobility reduction comes from Google COVID-19 Community Mobility Reports, which measure the percent change in mobility relative to pre-pandemic levels. Per capita GDP comes from the World Bank’s World Development Indicators; we use log per capita GDP. The Economic Support index from the Oxford COVID-19 Government Response tracker, which measures the extent of economic support across metrics such as income support and debt relief. We standardize all variables into z-scores—having a mean of zero and standard deviation of one. ‘N’ indicates the number of countries with available data. Maps produced using R, version 4.2.2 (https://www.r-project.org/); data for country boundaries come from Natural Earth (https://www.naturalearthdata.com/).

**Figure 6:**
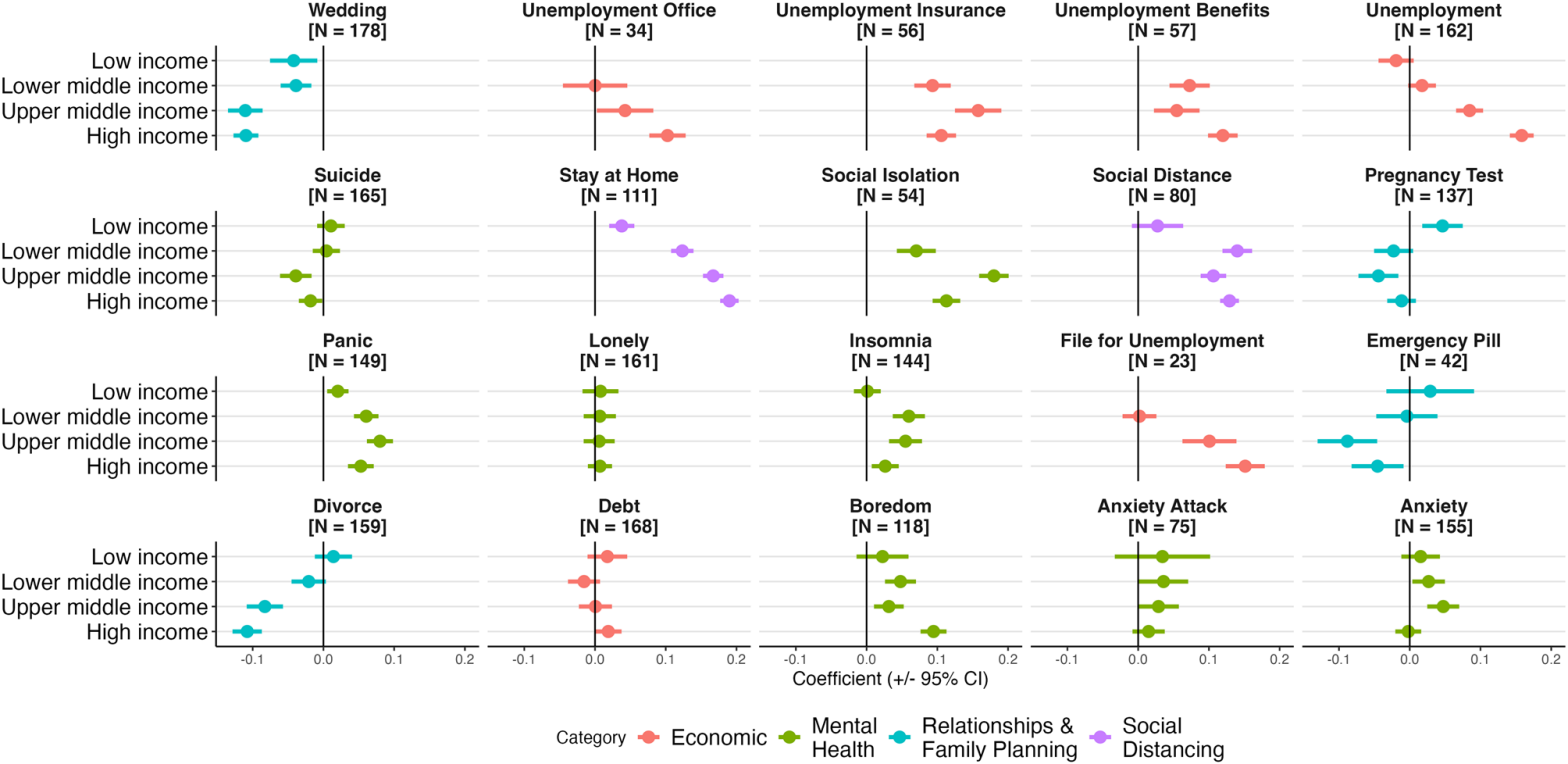
Association of COVID-19 policies and search interest: difference-in-difference results pooling countries by income level. Point estimates and 95% confidence intervals are shown. ‘N’ indicates the number of countries with available data.

The reactions to and the experience of the pandemic were not homogeneous across countries, however. We explore how the association of containment policies with search interest differed across countries along three dimensions: (1) income level, (2) the level of economic support provided to citizens, and (3) how restrictive containment policies were (proxied by the observed reduction in mobility and an index measuring the stringency of containment policies). Figure 5 shows results from difference-in-differences analysis, where we interact the difference-in-differences coefficient with indicators along each of the three dimensions. Figure 6 shows results from a differences-in-differences analysis where we separately run models for different country income groups. These results emphasize key trends.

First, countries with greater economic support saw higher search interest for select unemployment keywords after containment policies were implemented (Fig. 5). The mechanism for this result could be twofold: greater economic support may drive search interest in seeking economic support (for example, seeking unemployment benefits); alternatively, countries that provided more economic support may have experienced worse economic conditions which could drive search interest in unemployment and unemployment resources.

Second, countries with more restrictive containment policies saw (1) higher search interest in a number of mental health keywords, (2) higher search interest in “unemployment” (and other unemployment related keywords when using 30 and 60 day thresholds, see Appendix S13), and (3) lower search interest in relationship keywords after containment policies were implemented (Fig. 5). These results emphasize that countries with more restrictive containment policies experienced more pronounced economic, mental health, and social externalities.

Third, we find that higher-income countries saw a larger increase in social distancing and unemployment-related searches and a greater reduction in most relationship and family planning keywords relative to lower-income countries (Figs. 5, 6). In terms of the association between income level and the search for unemployment resources, the results could indicate that there were fewer unemployment resources in lower-income countries, and thus fewer resources to search for.

## Discussion

We demonstrate that Google search data can inform the monitoring and understanding of the COVID-19 pandemic at the global level using data across 221 countries. In particular, we show that Google search data can inform COVID-19 policies along two key dimensions: (1) capturing rising COVID-19 caseloads, where trends in search interest across select search terms often precede trends in cases; and (2) understanding how COVID-19 containment policies were associated with changes in social, economic and mental health indicators.

First, we find that search interest in symptoms that are more specific to COVID-19 (particularly, loss of smell and taste) were strongly correlated with COVID-19 cases throughout the world, especially in the first year of the pandemic; as Omicron became the dominant variant and loss of smell and taste became less common symptoms, the correlation between search interest in these terms and reported cases became notably lower. The correlation between search interest in “COVID symptoms” and reported cases remained strong throughout the pandemic. These results point to the importance of understanding the specific and evolving symptoms of a disease to be able to monitor searches related to such diseases, as well as exploring other search terms that may help to anticipate cases despite a change in the symptoms.

On average, search interest in loss of smell and taste is most highly correlated with COVID-19 cases following a lag of 11 days in 2020 and a lag of 3–6 days in 2021 and 2022, indicating that searches for COVID-specific symptoms tend to precede trends in COVID-19 cases—with a larger lag earlier on in the pandemic. This time lag provides valuable time to monitor a possible change in cases and anticipate new waves. This result highlights the potential of Google search data to support the monitoring and anticipation of COVID-19 cases throughout the world.

Importantly, we find that the capacity of Google search interest data to monitor and anticipate COVID-19 cases is not limited to high-income countries. On average, the results are as strong in lower-income countries as they are in higher-income countries. Considering the limited resources that developing countries often have to monitor the spread of the pandemic, the opportunities generated by Google search data are particularly promising for such countries.

Second, we find that internet searches are a valuable tool to understand the effects of COVID-19 shutdown policies—such as school closures, stay-at-home requirements, and travel restrictions—across the world. Pooling together all countries with available data and using a difference-in-differences approach, we find that containment policies were associated with an increase in search interest in keywords related to unemployment, mental health, and social distancing, and a reduction in search interest in keywords related to relationships and family planning. Our analysis also reveals important differences in the persistence of these effects: while select mental health terms (such as “panic” and “boredom”) returned to pre-pandemic levels relatively quickly, select economic indicators did not return to pre-pandemic levels until 2021 or 2022—highlighting the opportunities to distinguish the immediate from the long-term effects of the pandemic. Overall, the results emphasize the utility of search interest data to assess the social and economic repercussions of pandemic policies. As search interest data is available in real-time, the data could be used in future crises to understand impacts of crises and policy responses as they occur—indicating that search interest data could be a tool for informing policy responses.

Additionally, we find notable heterogeneity in the association of containment policies with changes in search interest across different levels of economic support and mobility restrictions. We find that countries with more restrictive containment policies saw higher search interest in many mental health keywords and in “unemployment”, and a reduction in search interest in select relationship and family planning keywords. Overall, the results suggest that countries with more restrictive policies experienced more pronounced economic, mental health, and social externalities.

Our analysis relies on country-level data; however, Google also enables querying subnational search interest data. Extending our analysis at the global, subnational level would be informative as many countries experienced heterogeneity in COVID-19 rates and responses to COVID-19^42–45^. We focus on country-level data as data on COVID-19 cases and other social and economic outcomes are more readily available at the country level. However, policymakers and practitioners may find it useful to track key Google Trends indicators at subnational levels too.

Relying on Google search interests comes with a set of limitations. For example, search interest can be driven by global news events rather than country-specific dynamics. Search interest serves as an imperfect—albeit often strong—proxy for on-the-ground conditions. However, a key advantage is that search interest data is free and immediately accessible in real-time. Moreover, our results emphasize that Google search data can help monitor the spread and consequences of the pandemic even in low-resource settings. By providing insights across multiple phases of the pandemic, our results demonstrate the potential of using the widespread expansion of technology to better understand and address pressing, real-time issues that affect citizens throughout the entire world.

## Methods

In this section, we first describe Google search interest data, which is used throughout the paper. We then describe the data and methodology used for each of the two sets of analyses: (1) search interest correlating with and preceding cases and (2) association of containment policies with search interest indicators.

### Google search interest data

Google search interest data was queried using the gTrendsR R package for the period between September 1, 2018 and December 31, 2022 at the daily level^10^. We query data across 221 countries and for 34 search terms related to COVID-19 symptoms and possible social, mental health, and economic consequences of the pandemic (Appendix S1 provides a list of search terms and the number of countries with data for each term).

For each country, we use a translated version of search terms using the language with the highest search activity in Google. We determine which language has the highest search activity by comparing search interest for “fever”, “doctor”, “hospital”, “food”, “restaurant”, and “football” across the most widely used languages in each country. Translations are done using the Google Translate API; Appendix S2 details the specific steps used to determine the language with the highest search activity in each country. An alternative approach could be to consider multiple languages, instead of the language with the highest search activity. We use only one language for each country for two reasons. First, the language with the highest search activity typically has notably higher search activity than the language with the second highest search activity. Across countries with two or more languages considered, the language with the highest search activity is about 100% more popular than the language with the second highest search activity in 75% of countries. Consequently, querying search interest across multiple languages and taking a weighted average based on the popularity of the language would only minimally change our results. Second, querying Google trends across multiple languages would significantly increase the number of queries that would need to be made to Google Trends. Google Trends limits the number of calls that can be made during a specific time period, so increasing the number of queries would notably increase the computation time for our global analysis.

Search interest information from Google reflects the absolute number of searches of a keyword or phrase relative to the total number of searches over a specific geographic area and period of time^46^. Consequently, the search interest indicator reflects the relative popularity of a keyword as opposed to the absolute number of searches. Google scales the search interest value so that the maximum value is 100 for a given query (e.g., search interest for “loss of smell” in the United States is scaled so that maximum value is 100). Google only provides search interest values if there is sufficient search activity; consequently, a search interest value of zero indicates that there could be some search interest for a search term, but there was not sufficient data.

One challenge of using Google Trends is that Google only provides daily values of search interest when 270 days or less are queried; given that Google scales values for each query between 0 and 100, the raw search interest values will not be comparable across queries of different date ranges. We create a consistent time series by querying search interest across time periods that overlap, then—relying on search interest in overlapping time periods—scale the data sets to create a consistent time series (see Appendix S3 for additional details). We use separate queries for each country and search term; consequently, the search interest data we query is only comparable across time within a specific country and keyword.

A second challenge of using Google Trends is that for a given time interval, Google Trends data is based on a random subsample drawn from Google^47^. Variation in Google Trends data resulting from this sampling can be large in cases of small countries/regions and for keywords with low search volume. Eichenauer et al.^47^ developed a solution to this problem, which relies on drawing multiple random samples of Google Trends data. Leveraging this approach would lead to the best quality Google Trends data; however, the approach requires a notably higher number of queries to Google Trends. Given that Google limits the number of queries that can be made in a specific time interval, using this approach is difficult in our context as we query over 30 keywords for over 200 countries—making the computational burden of using this approach non-trivial. Consequently, our approach of using Google Trends does not account for random sampling variation. This sampling variation likely adds noise to our data, and suggests that our results serve as a lower bound for the relation of Google Trends and other outcomes. Accounting for sampling variation—and reducing noise in the data—would likely improve results (e.g., the association between COVID-19 and reported search interest).

### Google search interest correlating and anticipating COVID-19 cases

The first set of analyses seeks to understand which search terms correlate with COVID-19 cases and whether trends in search interests precede trends in cases. Daily COVID-19 case data comes from the World Health Organization^48^.

For this analysis, we focus on keywords relating to the coronavirus generally (“coronavirus”, “COVID-19”, “how to treat coronavirus”), symptoms that are more common to COVID-19 (“loss of taste”, “loss of smell”, “I can’t smell”, “I can’t taste”) and more general symptoms and associated diseases (“fever”, “cough”, “tired”, “pneumonia”). For each country and keyword, we compute the correlation coefficient between search interest and cases to understand which search terms tend to see the highest correlations across countries. In addition, following^8,13,32,33,49–53^, we use a lag correlation analysis to understand whether search interest correlates more strongly with cases when using a lagged value of cases. We compute the correlation between search interest and cases by using lead and lag values of cases up to 21 days; the 21 day lead/lag follows from^50^. Search interest correlating most strongly with lagged cases would suggest that search interest picks up on trends in patterns before they are reflected in official data; search interest correlating most strongly with a lead (positive shift) in cases would suggest that search interest just reacts to on-the-ground conditions.

A more advanced model could be considered to estimate and predict reported COVID-19 cases; for example, Google Flu Trends—developed for early detection of influenza—considered 50 million search queries^54^, and other studies have used Google Flu Trends data and time-series models to enhance influenza prediction^55^. However, we leverage correlation analysis for two reasons. First, doing so aligns with similar literature that analyzes the association between Google search interest and reported COVID-19 cases. Second, correlation analysis that relies on single search terms (as opposed to an index) mimics the type of data a policymaker or practitioner would be able to readily retrieve from the Google Trends website. The Google Trends website makes it easy to see recent trends in individual search terms^2^. Consequently, while correlation analysis is relatively simple, showing which search terms are correlated with COVID-19 cases could help inform policymakers or practitioners about which search terms to track on the Google Trends website. While we do not implement a formal, predictive model, understanding that a certain search term tends to be correlated with COVID-19 cases—and seeing a sustained increase in search activity in that search term—could still provide useful insight to a policymaker or practitioner, suggesting that COVID-19 cases may be increasing or that the on-the-ground COVID-19 situation warrants further investigation.

A potential concern of using Google trends for evaluating the change in COVID-19 cases is that correlations may diminish over time; as the public’s understanding of COVID-19 increases over time, there may be less of a need to turn to Google during a rise in cases. Moreover, correlations may also change as the severity and main symptoms of COVID-19 changed with new variants and with increasing vaccination rates. To test how correlations may change over time, we compute the correlation using data from 2020, 2021, and 2022 separately.

We also examine potential factors that may explain where Google search interest provides the best proxy for COVID-19 cases. Here, we regress a set of country-level indicators on the correlation between COVID-19 cases and search interest in select keywords. Given the high correlation between loss of smell and reported cases, we focus on search interest in “loss of smell”. As the correlation between “loss of smell” and reported cases became lower in 2022, we use the correlation generated when using data from January 1, 2020 until December 31, 2021. For explanatory variables, we use the total number of COVID-19 cases as of December 31, 2021, metrics that capture online presence (percent of the population that uses internet and mobile cell subscribes per 100, both captured via the World Development Indicators, WDI, using data from 2019) and GDP per capita (also captured in the WDI). We estimate a similar regression using the lead/lag value that produces the highest correlation as the dependent variable. This analysis aims to understand which factors may increase or reduce the time lag between search interest and COVID-19 case reporting.

### Association of COVID-19 containment policies with social, mental health, and economic search interest indicators

Data on containment policies come from the University of Oxford COVID-19 Government Response Tracker dataset^56^. To understand the association of containment policies with search interest indicators, we rely on the first date that a country implemented any containment or closure policy, such as school closures, workplace closures, cancellation of public events, restrictions on gatherings, public transportation closures, stay at home requirements, restrictions on internal movements, and international travel controls. We examine how containment policies are associated with search interest for terms related to social distance measures (“social distance” and “stay at home”); mental health (“anxiety”, “anxiety attack”, “boredom”, “insomnia”, “lonely”, “panic”, “social isolation”, and “suicide”); relationship and family planning indicators (“divorce”, “wedding”, “emergency pill”, and “pregnancy test”); and economic, unemployment, and unemployment resource indicators (“debt”, “unemployment”, “file for unemployment”, “unemployment benefits”, “unemployment insurance”, and “unemployment office”). These search terms have been previously studied for a limited number of countries. Social distance measure keywords were informed by^57^, who examine trends across US states; relationship and family planning keywords were drawn from^23^, who examine trends in the United States and Europe; and mental health-related keywords largely come from^58^, who examine trends in select European countries. In this paper, we extend the analysis to all countries with available data, which makes it possible to compare the effects across countries with different containment policies. We aim to include search terms that would have broad relevance across countries; to this point, select search terms also appear in Demographic and Health Surveys (DHS)—which are a standardized set of surveys implemented in over 90 countries. For example, “divorce” and “emergency contraception”—similar to “emergency pill”—appear in DHS questionnaires, along with terms such as “anxiety” or “anxious”^59^.

We explore how the association of containment policies with search interest varies with the (1) restrictiveness of containment policies, (2) the level of government economic support, and (3) income level (GDP per capita). Specifically, we aim to understand whether the association of containment policies and search interest in economic and mental health keywords were larger in countries with more restrictive policies, whether economic support blunted any negative externalities of policies, and whether results vary systematically by income level. We use two datasets to measure the restrictiveness of containment policies. First, we use Google COVID-19 Community Mobility Reports, which measure the percent change in mobility relative to pre-pandemic levels to a number of types of locations^60^. We use the average percent change in mobility across retail and recreation locations, groceries and pharmacies, parks, transit stations and workplaces. Second, we use the Stringency Index from the University of Oxford COVID-19 Government Response tracker, a composite measure of the restrictiveness of nine policy measures, including workplace closures, public transport closures, and restrictions on public gathers, among other metrics^56^. To measure the level of government economic support, we use the Economic Support index from the Oxford COVID-19 Government Response tracker, which measures the extent of economic support across metrics such as income support and debt relief.

As our primary model, we implement a difference-in-differences model using data since the year before the beginning of the pandemic. We estimate the following model, using data from 90 days before and after the first containment policy in each country:

1 $$\begin{aligned} {y_{c,d,p}} & = {\beta _0} + {\beta _1} Post\ Policy\ {Period_{d}} \times Pandemic\ {Period_{y} } \\ & \quad+\beta _2 Post\ Policy\ {Period_{d}} \times Pandemic\ {Period_{y}} \times Country\ {Feature_{c} } \\ & \quad+ {\delta _c }+{ \gamma _d }+ {\epsilon _{c,d,p}} \end{aligned}$$

where $y_{c,d,p}$ is the search interest for country *c*, *d* days since the day and month the policy was implemented (irrespective of the year), and where *p* is the period (either pre-pandemic or pandemic period). *Post* *Policy* *Period* is a binary variable that turns on after the day and month the policy was implemented (irrespective of the year) and *Pandemic* *Period* is a binary variable that turns on in 2020. *Country* *Feature* is a country-level variable used to understand how the association of containment policies and search interest varies across countries; we use four features: (1) the Economic Support index, (2) the Stringency index, (3) mobility reduction, and (4) GDP per capita. We run separate regressions for each *Country* *Feature*. We scale all four features so that they have a mean of zero and standard deviation of one. For economic support, mobility reduction, and stringency index, we use the most extreme value in the 90-day period after the first containment policy was implemented. $\gamma$ are day of week fixed effects and $\delta$ are country fixed effects. In the regression, we also include all individual variables and two-way interactions of $Post~Policy~Period_{d} \times Pandemic~Period_{y} \times Country~Feature_{c}$. To test the sensitivity of results to the 90-day threshold, we also estimate models using 30, 60, 120 and 180-day thresholds.

To further test the sensitivity of results, we also estimate an event study approach that just relies on the time period from before and after policies were implemented. Here, we estimate the following model:

$$\begin{aligned} y_{i,t} = \beta _0&+ \sum _{f = -90}^{-2} \beta _f I(date - R_i = f) \\&+ \sum _{l = 0}^{90} \beta _l I(date - R_i = l) \\&+ \gamma _i + \delta _t + \epsilon _{i,t} \end{aligned}$$

where $y_{i,t}$ is the search interest value for a specific search term for country *i* on date t *t*, $R_i$ is the date when the first policy was implemented for country *i*. We subset the data to 90 days before and after the policy was implemented.

### Supplementary Information

Supplementary Information.

## Data Availability

The data and code used in this paper are available at https://github.com/worldbank/covid-gtrends. In addition, the data can be interactively explored in this dashboard: https://datanalytics.worldbank.org/covid_gtrends/.
