# Supplementary material for: The evolution of the COVID-19 pandemic through the lens of google searches
Source: Sci Rep. 2023 Nov 13;13:19843. doi: 10.1038/s41598-023-41675-4 (PMC10645993; doi:10.1038/s41598-023-41675-4)
Supplement: Supplementary file 1 — Supplementary Information. [file 41598_2023_41675_MOESM1_ESM.pdf]

The Evolution of the COVID-19 Pandemic Through the Lens of Google Searches

Supplementary Information

The findings, interpretations, and conclusions expressed in this paper are entirely those of the authors. They do not necessarily represent the views of the World Bank and its affiliated organizations, or those of the Executive Directors of the World Bank or the governments they represent.

Contents

S1 Search terms queried for analysis **S2**

S2 Determining language with most Google search activity in each country **S3**

S3 Create consistent time-series of Google search interest **S7**

S4 Correlation and lag between search interest and COVID-19 cases **S9**

S5 Map of correlations of search interest in "Loss of Smell" and "Fever" with COVID-19 cases **S14**

S6 Trends in search interest for "Loss of Smell" and COVID-19 cases for all countries with available data **S15**

S7 Trends in search interest for "COVID Symptoms" and COVID-19 cases for all countries with available data **S16**

S8 Trends in search interest for "Coronavirus" and COVID-19 cases for all countries with available data **S18**

S9 Correlation between search interest and reported COVID-19 cases by six months increments **S20**

S10 Explaining correlation between search interest and COVID-19 cases: additional results **S21**

S11 Comparing correlation results using reported COVID-19 cases vs excess mortality **S28**

S12 Association of containment policies with search interest: event study results **S37**

S13 Association of Containment Policies with Search Interest: Sensitivity Analysis Across Different Day Thresholds **S38**

S13.1 30 day threshold S38
S13.2 60 day threshold S41
S13.3 120 day threshold S44
S13.4 180 day threshold S47

S1 Search terms queried for analysis

Google only releases search information if there is sufficient data [2]. In this section we show the number of countries where there was sufficient Google search data for each search term, among countries with available COVID-19 case data. Table S1 provides information for search terms related to COVID-19 generally and to possible COVID-19 symptoms. Table S2 provides information for search terms used to understand the association containment policies with search interest.

Table S1: Symptoms Search Terms: Number of countries with available Google search interest data for each search term

Search Term	N Countries
Ageusia	86
Anosmia	108
COVID Symptoms	145
COVID-19	209
Coronavirus	212
Cough	197
Fever	205
How to Treat Coronavirus	61
I Can't Smell	34
I Can't Taste	17
Loss of Smell	112
Loss of Taste	105
Pneumonia	180
Shortness of Breath	132

Table S2: Containment Policy Analysis Search Terms: Number of countries with available Google search interest data for each search term

Search Term	N Countries
Anxiety	181
Anxiety Attack	77
Boredom	120
Debt	191
Divorce	189
Emergency Pill	46
File for Unemployment	27
Insomnia	159
Lonely	184
Panic	168
Pregnancy Test	157
Social Distance	89
Social Isolation	57
Stay at Home	122
Suicide	197
Unemployment	188
Unemployment Benefits	62
Unemployment Insurance	55
Unemployment Office	38
Wedding	214

S2 Determining language with most Google search activity in each country

We use the following steps to determine the language with the highest Google search activity for each country:

- **Step 1:** For each search term t, we query search interest across translated versions of the search term within the same query. We query weekly search interest for all of 2020. The translated versions of search term t are included in the same query, which ensures search interest values across the translated terms are comparable.
- **Step 2:** The above step produces search interest at the weekly level for each translated version of search term t. We take the average value across the time series, yielding one value per translated version of search term t.
- **Step 3:** For each search term t, we standardize values of search interest for translated terms so that the maximum value is 100. This step is done because, from the above step, values are only comparable within translated versions of search term t.
- **Step 4:** Across languages, take the average value from the above step. The language with the highest value is used at the language with the highest search interest.

Table S3 shows an illustrative example using a country where English, French and Spanish are the main languages spoken, where we just consider translations for three words: "fever", "doctor" and "hospital"; in practice, we also use "food", "restaurant" and "football." The below example highlights the benefit of querying multiple search terms; the words for "doctor" and "hospital" are the same in English and Spanish, while the world for "fever" differs in English and Spanish. In addition, French sees the highest search activity for "fever" and "doctor" compared to English and Spanish versions; however, the French version of "hospital" sees slightly lower interest than the English and Spanish versions. Considering all search terms, though, indicates French will typically capture high search activity compared to searches in other languages. In step 3, standardizing allows comparing values between different search search terms; and averaging these values in step 4 shows that French has the highest average search activity; consequently, for this country, we query Google search interest in French.

Table S3: Illustrative example of determining most common language for Google searches

	Fever			Doctor			Hospital		
	English	French	Spanish	English	French	Spanish	English	French	Spanish
	Fever	fièvre	fiebre	doctor	médecin	doctor	hospital	hôpital	hospital
**Step 1:** Query weekly search interest for each translated search term									
Week 1	10	50	5	20	80	20	100	90	100
Week 2	50	100	10	10	75	10	60	70	60
Week 3	30	60	15	15	100	15	50	20	50
**Step 2:** Take average values across time series									
Avg	30	70	10	15	85	15	70	60	70
**Step 3:** Standardize values within translated search terms so maximum value is 100									
	42.85	100	14.28	17.64	100	17.64	100	85.71	100
**Step 4:** Average value across languages; French is used as it has the highest value below.									
• English: (42.85 + 17.64 + 100)/3 = 53.49									
• French: (100 + 100 + 85.71)/3 = 95.23									
• Spanish: (14.28 + 17.64 + 100)/ = 43.97									

Table S4 shows, for each country, the language with the highest Google search activity and the average search activity (S.A.) for each language considered. For each country, we compare search interest in languages identified here: `https://github.com/annexare/Countries/blob/master/dist/countries.csv`.

Table S4: Google search activity across countries and languages

Country	Language with Highest S.A.	Language and Search Activity (S.A.)				
		Name (S.A.)	Name (S.A.)	Name (S.A.)	Name (S.A.)	Name (S.A.)
Andorra	Catalan	Catalan (100)				
United Arab Emirates	Arabic	Arabic (100)				
Afghanistan	Pashto	Pashto (75)	Turkmen (25)	Uzbek (25)		
Antigua & Barbuda	English	English (100)				
Anguilla	English	English (100)				
Albania	Albanian	Albanian (100)				
Armenia	Russian	Armenian (69.5)	Russian (76.2)			
Angola	Portuguese	Portuguese (100)				
Argentina	Spanish	Spanish (100)				
American Samoa	English	English (99.7)	Samoan (14.3)			
Austria	German	German (100)				
Australia	English	English (100)				
Aruba	Dutch	Dutch (100)	Punjabi (0)			
Azerbaijan	Azerbaijani	Azerbaijani (100)				
Bosnia & Herzegovina	Bosnian	Bosnian (93.4)	Croatian (81.7)	Serbian (0.1)		
Barbados	English	English (100)				
Bangladesh	Bengali	Bengali (100)				
Belgium	Dutch	German (16.6)	French (53.9)	Dutch (78.1)		
Burkina Faso	French	French (100)				
Bulgaria	Bulgarian	Bulgarian (100)				
Bahrain	Arabic	Arabic (100)				
Burundi	French	French (100)				
Benin	French	French (100)				
St. Barthélemy	French	French (100)				
Bermuda	English	English (100)				
Brunei	Malay	Malay (100)				
Bolivia	Spanish	Spanish (100)				
Brazil	Portuguese	Portuguese (100)				
Bahamas	English	English (100)				
Botswana	English	English (100)				
Belarus	Russian	Belarusian (15.4)	Russian (100)			
Belize	English	English (100)	Spanish (35.5)			
Canada	English	English (100)	French (30.5)			
Congo - Kinshasa	French	French (100)	Swahili (0.8)			
Central African Republic	French	French (100)				
Congo - Brazzaville	French	French (100)				
Switzerland	German	German (86.7)	French (51.7)	Italian (30.4)		
Côte d'Ivoire	French	French (100)				
Cook Islands	English	English (100)				
Chile	Spanish	Spanish (100)				
Cameroon	English	English (97.9)	French (64.5)			
China	Chinese	Chinese (100)				
Colombia	Spanish	Spanish (100)				
Costa Rica	Spanish	Spanish (100)				
Cuba	Spanish	Spanish (100)				
Cape Verde	Portuguese	Portuguese (100)				
Curaçao	English	English (88.9)	Dutch (67.4)	Punjabi (0)		
Cyprus	Turkish	Greek (36)	Armenian (0)	Turkish (94.5)		
Czechia	Czech	Czech (100)	Slovak (0.7)			
Germany	German	German (100)				
Djibouti	French	Arabic (2.5)	French (100)	Somali (0)		
Denmark	Danish	Danish (100)				
Dominica	English	English (100)				
Dominican Republic	Spanish	Spanish (100)				
Algeria	Arabic	Arabic (100)				
Ecuador	Spanish	Spanish (100)				
Estonia	Estonian	Estonian (100)				
Egypt	Arabic	Arabic (100)				
Eritrea	English	Arabic (0)	English (100)			
Spain	Spanish	Catalan (46.6)	Spanish (85.8)	Basque (0.3)	Galician (47.5)	
Ethiopia	Amharic	Amharic (100)				
Finland	Finnish	Finnish (100)	Swedish (13.5)			
Fiji	English	English (100)	Hindi (0)	Urdu (0)		
Falkland Islands	English	English (100)				
Micronesia	English	English (100)				
France	French	French (100)				
Gabon	French	French (100)				
United Kingdom	English	English (100)				
Grenada	English	English (100)				
Georgia	Georgian	Georgian (100)				
French Guiana	French	French (100)				

Guernsey	English	English (100)	French (28.6)		
Ghana	English	English (100)			
Gibraltar	English	English (100)			
Greenland	Danish	Danish (100)			
Gambia	English	English (100)			
Guinea	French	French (100)			
Guadeloupe	French	French (100)			
Equatorial Guinea	Spanish	Spanish (85.7)	French (25.9)		
Greece	Greek	Greek (100)			
Guatemala	Spanish	Spanish (100)			
Guam	English	English (100)	Spanish (28.6)		
Guinea-Bissau	Portuguese	Portuguese (100)			
Guyana	English	English (100)			
Hong Kong SAR China	English	English (83.9)	Chinese (49.3)		
Honduras	Spanish	Spanish (100)			
Croatia	Croatian	Croatian (100)			
Haiti	French	French (86.3)	Haitian Creole (34)		
Hungary	Hungarian	Hungarian (100)			
Indonesia	Indonesian	Indonesian (100)			
Ireland	English	English (100)	Irish (0.5)		
Israel	Hebrew	Arabic (44.9)	Hebrew (57.1)		
Isle of Man	English	English (100)			
India	English	English (100)	Hindi (5.7)		
Iraq	Arabic	Arabic (100)	Kurdish (0.9)		
Iran	Persian	Persian (100)			
Iceland	Icelandic	Icelandic (100)			
Italy	Italian	Italian (100)			
Jersey	English	English (100)	French (28.6)		
Jamaica	English	English (100)			
Jordan	Arabic	Arabic (100)			
Japan	Japanese	Japanese (100)			
Kenya	English	English (100)	Swahili (5.6)		
Kyrgyzstan	Russian	Kyrgyz (35.8)	Russian (100)		
Cambodia	Khmer	Khmer (100)			
Kiribati	English	English (100)			
Comoros	French	Arabic (0)	French (100)		
St. Kitts & Nevis	English	English (100)			
North Korea	Korean	Korean (100)			
South Korea	Korean	Korean (100)			
Kuwait	Arabic	Arabic (100)			
Cayman Islands	English	English (100)			
Kazakhstan	Russian	Kazakh (21.4)	Russian (100)		
Laos	Lao	Lao (100)			
Lebanon	Arabic	Arabic (85.3)	French (41.2)		
St. Lucia	English	English (100)			
Liechtenstein	German	German (100)			
Sri Lanka	Sinhala	Sinhala (83.3)	Tamil (42.7)		
Liberia	English	English (100)			
Lesotho	English	English (100)	Sesotho (1.7)		
Lithuania	Lithuanian	Lithuanian (100)			
Luxembourg	German	German (85.1)	French (66.8)	Luxembourgish (24.9)	
Latvia	Latvian	Latvian (100)			
Libya	Arabic	Arabic (100)			
Morocco	Arabic	Arabic (100)			
Monaco	French	French (100)			
Moldova	Romanian	Romanian (100)			
Montenegro	Bosnian	Bosnian (85.7)	Croatian (73.9)	Albanian (14.5)	Serbian (1)
St. Martin (French)	English	English (100)	French (33.3)	Dutch (16.7)	
Madagascar	French	French (88.3)	Malagasy (51.3)		
Marshall Islands	English	English (100)			
North Macedonia	Macedonian	Macedonian (100)			
Mali	French	French (100)			
Myanmar (Burma)	Myanmar	Myanmar (100)			
Mongolia	Mongolian	Mongolian (100)			
Macao SAR China	Chinese	Portuguese (18.8)	Chinese (99.3)		
Northern Mariana Islands	English	English (100)			
Martinique	French	French (100)			
Mauritania	Arabic	Arabic (100)			
Montserrat	English	English (100)			
Malta	English	English (100)	Maltese (1.3)		
Mauritius	English	English (100)			
Maldives	English	Arabic (0.1)	English (100)		
Malawi	English	English (100)	Nyanja (0)		
Mexico	Spanish	Spanish (100)			
Malaysia	Malay	Malay (100)			
Mozambique	Portuguese	Portuguese (100)			
Namibia	English	Afrikaans (18.3)	English (100)		
New Caledonia	French	French (100)			
Niger	French	French (100)			
Nigeria	English	English (100)			
Nicaragua	Spanish	Spanish (100)			

Netherlands	Dutch	Dutch (100)				
Norway	Norwegian	Norwegian (100)				
Nepal	Nepali	Nepali (100)				
Nauru	English	English (100)				
Niue	English	English (100)				
New Zealand	English	English (100)	Maori (3.3)			
Oman	Arabic	Arabic (100)				
Panama	Spanish	Spanish (100)				
Peru	Spanish	Spanish (100)				
French Polynesia	French	French (100)				
Papua New Guinea	English	English (100)				
Philippines	English	English (100)				
Pakistan	English	English (100)	Urdu (1)			
Poland	Polish	Polish (100)				
St. Pierre & Miquelon	French	French (100)				
Pitcairn Islands	English	English (100)				
Puerto Rico	English	English (84.1)	Spanish (79.7)			
Palestinian Territories	Arabic	Arabic (100)				
Portugal	Portuguese	Portuguese (100)				
Palau	English	English (100)				
Paraguay	Spanish	Spanish (100)				
Qatar	Arabic	Arabic (100)				
Réunion	French	French (100)				
Romania	Romanian	Romanian (100)				
Serbia	Serbian	Serbian (100)				
Russia	Russian	Russian (100)				
Rwanda	English	English (100)	French (29.9)	Kinyarwanda (17.3)		
Saudi Arabia	Arabic	Arabic (100)				
Solomon Islands	English	English (100)				
Seychelles	English	English (100)	French (28.6)			
Sudan	Arabic	Arabic (76.1)	English (68.4)			
Sweden	Swedish	Swedish (100)				
Singapore	English	English (100)	Malay (15.8)	Tamil (0)	Chinese (7)	
St. Helena	English	English (100)				
Slovenia	Slovenian	Slovenian (100)				
Slovakia	Slovak	Slovak (100)				
Sierra Leone	English	English (100)				
San Marino	Italian	Italian (100)				
Senegal	French	French (100)				
Somalia	English	Arabic (4.8)	English (100)	Somali (1.3)		
Suriname	Dutch	Dutch (100)				
South Sudan	English	English (100)				
São Tomé & Príncipe	Portuguese	Portuguese (100)				
El Salvador	Spanish	Spanish (100)				
Sint Maarten	English	English (100)	Dutch (14.3)			
Syria	Arabic	Arabic (100)				
Eswatini	English	English (100)				
Turks & Caicos Islands	English	English (100)				
Chad	French	Arabic (0.8)	French (100)			
Togo	French	French (100)				
Thailand	Thai	Thai (100)				
Tajikistan	Russian	Russian (100)	Tajik (20.5)			
Tokelau	English	English (100)				
Timor-Leste	Portuguese	Portuguese (100)				
Turkmenistan	Russian	Russian (99.6)	Turkmen (29.9)			
Tunisia	Arabic	Arabic (100)				
Tonga	English	English (100)				
Turkey	Turkish	Turkish (100)				
Trinidad & Tobago	English	English (100)				
Tuvalu	English	English (100)				
Taiwan	Chinese	Chinese (100)				
Tanzania	English	English (100)	Swahili (22.3)			
Ukraine	Ukrainian	Ukrainian (100)				
Uganda	English	English (100)	Swahili (0.3)			
United States	English	English (100)				
Uruguay	Spanish	Spanish (100)				
Uzbekistan	Russian	Russian (87.9)	Uzbek (44.2)			
St. Vincent & Grenadines	English	English (100)				
Venezuela	Spanish	Spanish (100)				
British Virgin Islands	English	English (100)				
U.S. Virgin Islands	English	English (100)				
Vietnam	Vietnamese	Vietnamese (100)				
Vanuatu	English	English (100)	French (28.6)			
Wallis & Futuna	French	French (100)				
Samoa	English	English (100)	Samoan (0.5)			
Yemen	Arabic	Arabic (100)				
Mayotte	French	French (100)				
South Africa	English	Afrikaans (17.6)	English (100)	Sesotho (0.2)	Xhosa (0)	Zulu (0.1)
Zambia	English	English (100)				
Zimbabwe	English	English (100)	Shona (0.5)			

S3 Create consistent time-series of Google search interest

Google trends provides a scaled index of search interest that ranges between 0 and 100 across a specific time range and geographic region [2]. Google only allows querying data for a time range of up to 270 days; consequently, to create a consistent time series from September 1, 2018 to December 31, 2022, we query data for nine different time ranges. Given that Google scales values between 0 and 100 for each query, the values across different the different time series queries will not be comparable.

To create a consistent time series, we query data across time ranges that overlap. Specifically, we query data for:

- September 1, 2018 - May 28, 2019
- January 1, 2019 - September 27, 2019
- July 1, 2019 - March 26, 2020
- January 1, 2020 - September 26, 2020
- July 5, 2020 - March 31, 2021
- January 4, 2021 - September 30, 2021
- April 6, 2021 - December 31, 2021
- October 1, 2021 - June 27, 2021
- April 6, 2022 - December 31, 2022

We then use search interest in the overlapping time periods to scale the full range of data so that the range of values within overlapping time spans are the same. Specifically, following [3], we use the below equation:

$$\frac{m - r_{min}}{r_{max} - r_{min}} \times (t_{max} - t_{min}) + t_{min} \quad (1)$$

where:

- r_{min} is the minimum value from the values to be scaled
- r_{max} is the maximum value from the values to be scaled
- t_{min} is the minimum of the target scaling
- t_{max} is the minimum of the target scaling
- $m \in [r_{min}, r_{max}]$ is the value to be scaled

and where r_{min}, r_{max}, t_{min}, t_{max} are determined from the set of values from the two time series where the dates overlap, and m is the full range of search interest values from the query. Figure S1 shows an example using search interest for "fever" in the United States; the figure shows both the raw values from the original search interest query (panel A) and the resulting consistent time series (panel B).

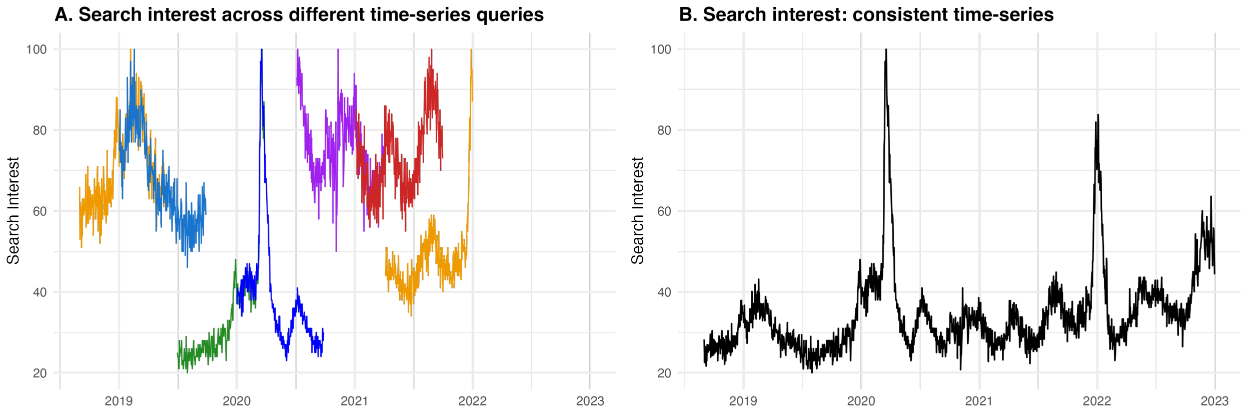

Figure S1: Creating a consistent time series of google search interest for search interest in "fever" for the United States. **Panel A** shows the raw search interest values across queries for search interest across different date ranges. Each query is scaled so its maximum value is 100. **Panel B** shows a consistent time series, creating from scaling values

S4 Correlation and lag between search interest and COVID-19 cases

Tables S5-S7 summarize the distribution of the correlation between search interest and COVID-19 cases across countries as well as the distribution of the lag of COVID-19 cases that resulted in the highest correlation with search interest. The separate tables summarize data for each year (2020, 2021, and 2022). Figure S2 compares the distribution of the original correlation and the correlation when using the optimal lag of COVID-19 cases; the correlations using the best lag are mechanically higher, although the distributions are generally similar.

Table S5: Correlation between search interest and COVID-19 cases using data in 2020

Term	Percentile							
	Min	5th	25th	50th	75th	95th	Max	N
**Correlation**								
Ageusia	-0.17	-0.08	-0.02	0.03	0.1	0.24	0.32	78
I Can't Taste	-0.1	-0.05	-0.01	0.06	0.12	0.31	0.6	15
How to Treat Coronavirus	-0.29	-0.2	-0.06	-0.02	0.09	0.23	0.34	42
Anosmia	-0.08	-0.04	0	0.06	0.14	0.28	0.43	98
Shortness of Breath	-0.25	-0.1	-0.03	0.02	0.07	0.19	0.33	128
I Can't Smell	-0.07	-0.06	-0.01	0.08	0.16	0.32	0.42	33
Cough	-0.46	-0.24	-0.07	-0.01	0.05	0.17	0.36	188
Pneumonia	-0.37	-0.18	-0.05	-0.02	0.06	0.33	0.63	173
Fever	-0.46	-0.28	-0.06	-0.01	0.06	0.18	0.41	197
Loss of Taste	-0.05	-0.04	0.05	0.13	0.25	0.6	0.83	99
Loss of Smell	-0.06	-0.04	0.06	0.17	0.33	0.64	0.81	104
Covid-19	-0.3	-0.13	-0.03	0.02	0.11	0.26	0.7	205
Coronavirus	-0.49	-0.39	-0.24	-0.12	-0.03	0.16	0.59	207
Covid Symptoms	-0.08	-0.04	0.02	0.11	0.3	0.62	0.72	136
**Correlation using best lag**								
Ageusia	-0.12	-0.05	0.02	0.12	0.2	0.35	0.53	78
I Can't Taste	-0.06	0.01	0.08	0.1	0.2	0.35	0.6	15
How to Treat Coronavirus	-0.22	-0.16	-0.03	0.05	0.23	0.4	0.51	42
Anosmia	-0.05	-0.02	0.07	0.16	0.24	0.43	0.52	98
Shortness of Breath	-0.15	-0.05	0.03	0.1	0.16	0.31	0.49	128
I Can't Smell	-0.06	-0.04	0.06	0.13	0.26	0.41	0.55	33
Cough	-0.41	-0.19	-0.03	0.06	0.13	0.29	0.45	188
Pneumonia	-0.35	-0.13	-0.01	0.08	0.19	0.4	0.74	173
Fever	-0.37	-0.2	0	0.08	0.17	0.33	0.73	197
Loss of Taste	-0.04	-0.01	0.11	0.25	0.37	0.66	0.86	99
Loss of Smell	-0.06	-0.02	0.17	0.28	0.43	0.68	0.88	104
Covid-19	-0.26	-0.06	0.04	0.15	0.22	0.47	0.75	205
Coronavirus	-0.47	-0.35	-0.17	-0.05	0.07	0.39	0.76	207
Covid Symptoms	-0.06	-0.01	0.14	0.24	0.41	0.69	0.9	136
**Lag with best correlation**								
Ageusia	-21	-21	-17.75	-10.5	5	21	21	78
I Can't Taste	-21	-20.3	-18.5	-16	0	20.3	21	15
How to Treat Coronavirus	-21	-20.95	-19	-16	-10	0.9	13	42
Anosmia	-21	-21	-17	-11	-4.25	18.3	21	98
Shortness of Breath	-21	-20	-16	-6	7.25	17	21	128
I Can't Smell	-20	-20	-16	-8	2	18.4	21	33
Cough	-21	-21	-14.25	-6.5	4.25	20	21	188
Pneumonia	-21	-21	-15	-6	6	19	21	173
Fever	-21	-21	-16	-7	4	19.2	21	197
Loss of Taste	-21	-21	-18	-11	-3.5	13.4	20	99
Loss of Smell	-21	-21	-18.25	-11	-2	16.85	21	104
Covid-19	-21	-21	-18	-11	-4	13.8	21	205
Coronavirus	-21	-21	-21	-16	-9	3	21	207
Covid Symptoms	-21	-21	-17	-10.5	-3.75	13.25	20	136

Table S6: Correlation between search interest and COVID-19 cases using data in 2021

Term	Percentile							
	Min	5th	25th	50th	75th	95th	Max	N
**Correlation**								
Ageusia	-0.22	-0.09	-0.03	0.02	0.09	0.19	0.34	76
I Can't Taste	-0.06	-0.05	-0.01	0.07	0.15	0.26	0.26	17
How to Treat Coronavirus	-0.21	-0.16	-0.05	0.08	0.17	0.29	0.32	58
Anosmia	-0.11	-0.06	0	0.04	0.13	0.26	0.52	100
Shortness of Breath	-0.15	-0.07	-0.02	0.04	0.12	0.36	0.87	123
I Can't Smell	-0.19	-0.11	-0.01	0.03	0.09	0.25	0.55	31
Cough	-0.47	-0.08	-0.01	0.06	0.15	0.39	0.9	188
Pneumonia	-0.19	-0.07	-0.01	0.05	0.15	0.44	0.94	168
Fever	-0.18	-0.07	-0.01	0.05	0.14	0.51	0.88	201
Loss of Taste	-0.15	-0.05	0.02	0.09	0.17	0.43	0.82	99
Loss of Smell	-0.05	-0.03	0.03	0.09	0.2	0.47	0.86	104
Covid-19	-0.16	-0.06	0	0.07	0.18	0.4	0.65	202
Coronavirus	-0.28	-0.1	-0.01	0.05	0.24	0.56	0.81	211
Covid Symptoms	-0.11	-0.05	0.04	0.13	0.29	0.67	0.94	138
**Correlation using best lag**								
Ageusia	-0.17	-0.01	0.03	0.1	0.18	0.36	0.48	76
I Can't Taste	0	0.01	0.09	0.14	0.24	0.29	0.39	17
How to Treat Coronavirus	-0.18	-0.14	0.04	0.14	0.27	0.34	0.51	58
Anosmia	-0.09	-0.03	0.08	0.13	0.22	0.34	0.56	100
Shortness of Breath	-0.09	-0.03	0.07	0.13	0.21	0.43	0.88	123
I Can't Smell	-0.1	-0.06	0.1	0.13	0.18	0.29	0.71	31
Cough	-0.38	-0.03	0.08	0.15	0.24	0.53	0.92	188
Pneumonia	-0.16	-0.01	0.08	0.16	0.25	0.57	0.95	168
Fever	-0.16	-0.01	0.09	0.14	0.24	0.62	0.91	201
Loss of Taste	-0.07	0	0.1	0.16	0.25	0.51	0.9	99
Loss of Smell	-0.02	0.02	0.11	0.2	0.28	0.56	0.94	104
Covid-19	-0.08	-0.01	0.1	0.17	0.29	0.51	0.76	202
Coronavirus	-0.21	-0.03	0.09	0.18	0.33	0.61	0.87	211
Covid Symptoms	-0.09	0.02	0.17	0.24	0.4	0.74	0.98	138
**Lag with best correlation**								
Ageusia	-21	-21	-15.25	0	17.25	21	21	76
I Can't Taste	-21	-19.4	-16	-9	3	17	21	17
How to Treat Coronavirus	-21	-21	-17.75	-11	2	20.15	21	58
Anosmia	-21	-21	-16	-4	12	20	21	100
Shortness of Breath	-21	-20	-10	1	13	20	21	123
I Can't Smell	-21	-21	-17.5	-5	12.5	21	21	31
Cough	-21	-21	-13	-4	3	19	21	188
Pneumonia	-21	-19	-11	-2	10	21	21	168
Fever	-21	-20	-12	-3	7	20	21	201
Loss of Taste	-21	-20	-11	-3	8.5	19.1	21	99
Loss of Smell	-21	-20	-14	-6	3	16.85	21	104
Covid-19	-21	-19	-12	-2	7	19.95	21	202
Coronavirus	-21	-20	-9	-1	10	20.5	21	211
Covid Symptoms	-21	-17	-10.75	-4	1.75	18.15	21	138

Table S7: Correlation between search interest and COVID-19 cases using data in 2022

Term	Percentile							
	Min	5th	25th	50th	75th	95th	Max	N
**Correlation**								
Ageusia	-0.11	-0.09	-0.04	0.02	0.07	0.15	0.28	62
I Can't Taste	-0.11	-0.11	-0.01	0.02	0.06	0.23	0.39	14
How to Treat Coronavirus	-0.13	-0.04	0	0.04	0.11	0.16	0.26	48
Anosmia	-0.1	-0.07	-0.02	0.03	0.11	0.26	0.49	89
Shortness of Breath	-0.14	-0.08	-0.03	0.03	0.11	0.29	0.54	115
I Can't Smell	-0.06	-0.05	-0.02	0.02	0.07	0.34	0.42	23
Cough	-0.29	-0.08	-0.01	0.05	0.13	0.47	0.77	187
Pneumonia	-0.21	-0.09	-0.03	0.02	0.09	0.26	0.55	158
Fever	-0.25	-0.08	-0.02	0.04	0.13	0.47	0.78	196
Loss of Taste	-0.1	-0.08	0	0.06	0.11	0.33	0.78	80
Loss of Smell	-0.09	-0.05	-0.01	0.03	0.12	0.4	0.72	85
Covid-19	-0.24	-0.05	0.02	0.13	0.34	0.71	0.91	198
Coronavirus	-0.2	-0.06	0.02	0.13	0.45	0.76	0.92	198
Covid Symptoms	-0.1	-0.06	0	0.1	0.27	0.74	0.86	138
**Correlation using best lag**								
Ageusia	-0.09	-0.03	0.04	0.09	0.16	0.26	0.3	62
I Can't Taste	-0.07	-0.02	0.04	0.1	0.16	0.27	0.43	14
How to Treat Coronavirus	0.02	0.04	0.07	0.14	0.18	0.26	0.38	48
Anosmia	-0.03	0.01	0.07	0.14	0.22	0.35	0.57	89
Shortness of Breath	-0.13	0.02	0.08	0.13	0.21	0.37	0.65	115
I Can't Smell	-0.01	0	0.09	0.13	0.18	0.41	0.43	23
Cough	-0.25	-0.03	0.08	0.15	0.26	0.61	0.88	187
Pneumonia	-0.17	-0.01	0.08	0.14	0.2	0.38	0.75	158
Fever	-0.19	0.01	0.09	0.15	0.26	0.64	0.89	196
Loss of Taste	-0.07	0	0.08	0.13	0.18	0.49	0.85	80
Loss of Smell	-0.02	0.01	0.08	0.12	0.22	0.48	0.83	85
Covid-19	-0.19	0.07	0.13	0.24	0.45	0.83	0.92	198
Coronavirus	-0.19	0.06	0.15	0.23	0.51	0.84	0.93	198
Covid Symptoms	-0.03	0.04	0.12	0.22	0.4	0.85	0.97	138
**Lag with best correlation**								
Ageusia	-21	-21	-13.5	0	11	20	21	62
I Can't Taste	-21	-19.05	-7.25	-4	6.5	16.75	20	14
How to Treat Coronavirus	-21	-20.65	-13.5	0	8.5	16	21	48
Anosmia	-21	-21	-13	-5	5	19.6	21	89
Shortness of Breath	-21	-20	-12	0	12	20	21	115
I Can't Smell	-21	-21	-17	-7	8	16.9	20	23
Cough	-21	-21	-15	-6	4	18	21	187
Pneumonia	-21	-21	-16	-3	11	20	21	158
Fever	-21	-20	-15	-8	4	19.25	21	196
Loss of Taste	-21	-21	-15	-6.5	5.25	18.05	21	80
Loss of Smell	-20	-18.8	-12	-3	11	18	21	85
Covid-19	-21	-19	-14	-5	5	17.15	21	198
Coronavirus	-21	-21	-15	-8	0	16	21	198
Covid Symptoms	-21	-20	-13	-5	3.75	17.15	21	138

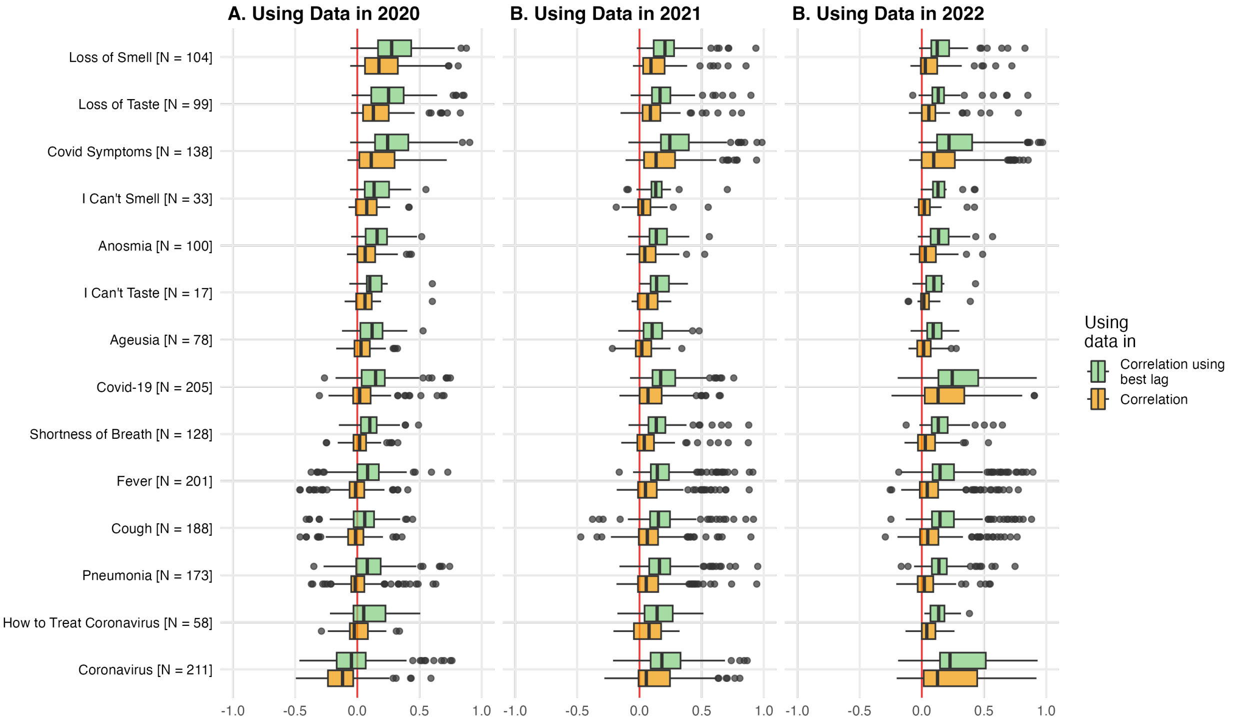

Figure S2: Distribution of correlation between search interest and COVID-19 cases using the original correlation and the correlation when using the lagged value of COVID-19 cases that produced the highest correlation. 'N' indicates the number of countries with available data. The boxplots include: center line, median; box limits, upper and lower quartiles; whiskers, 1.5x interquartile range; points beyond whiskers, outliers.

S5 Map of correlations of search interest in "Loss of Smell" and "Fever" with COVID-19 cases

Figure S3 shows a map of the correlations using "Loss of Smell" and a more general symptom ("Fever"); the figure illustrates how the correlation between COVID-19 cases and search interest in "Loss of Smell" is strong throughout different geographic regions. In addition, the figure illustrates that while search interest in more general symptoms such as "Fever" tend to have a lower correlation, these terms tend to have data across more countries—where in some countries more general search terms like "Fever" are highly correlated with reported COVID-19 cases.

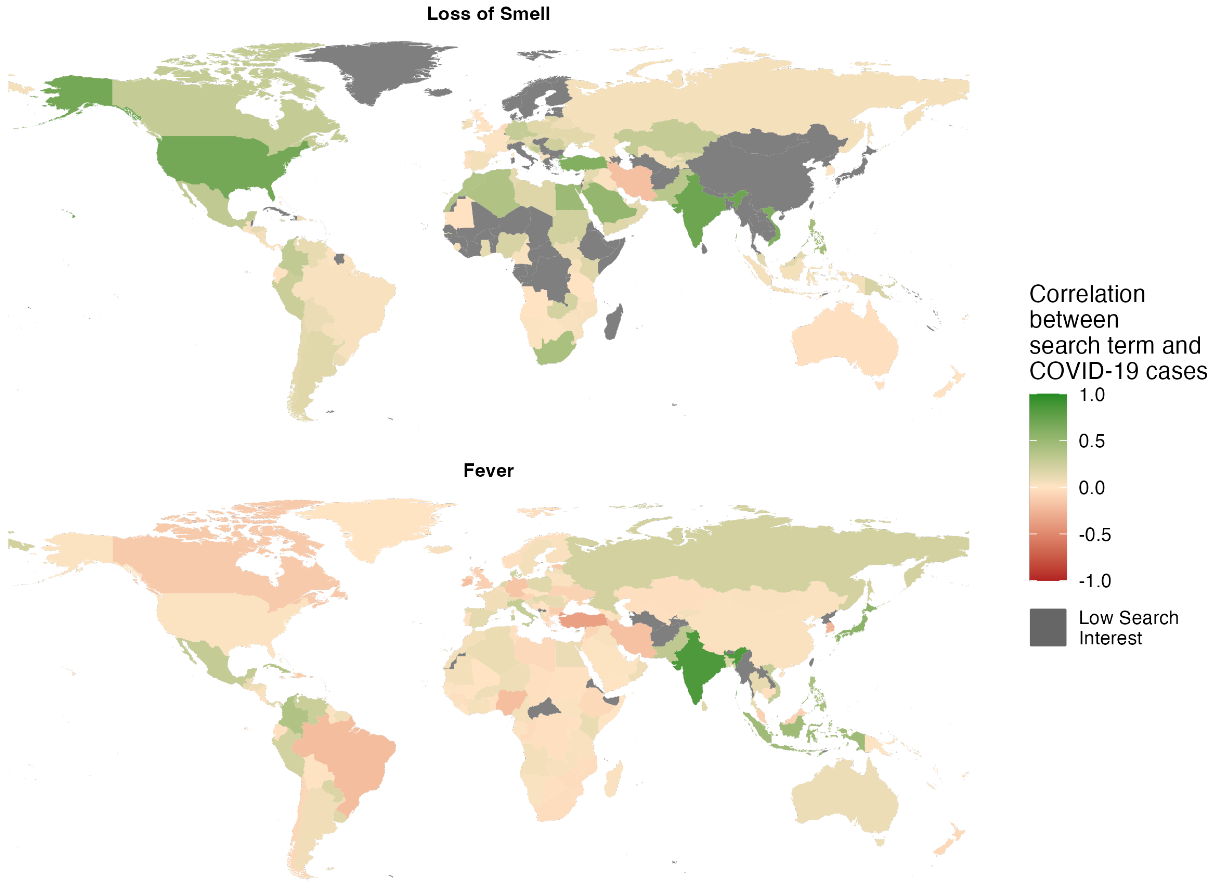

Figure S3: Correlation between reported COVID-19 cases and search interest in "Loss of Smell" and "Fever." Maps produced using R, version 4.2.2 (`https://www.r-project.org/`); data for country boundaries come from Natural Earth (`https://www.naturalearthdata.com/`).

S6 Trends in search interest for "Loss of Smell" and COVID-19 cases for all countries with available data

Figure S4 shows trends in search interest in "Loss of Smell" and COVID-19 cases for all countries with available data.

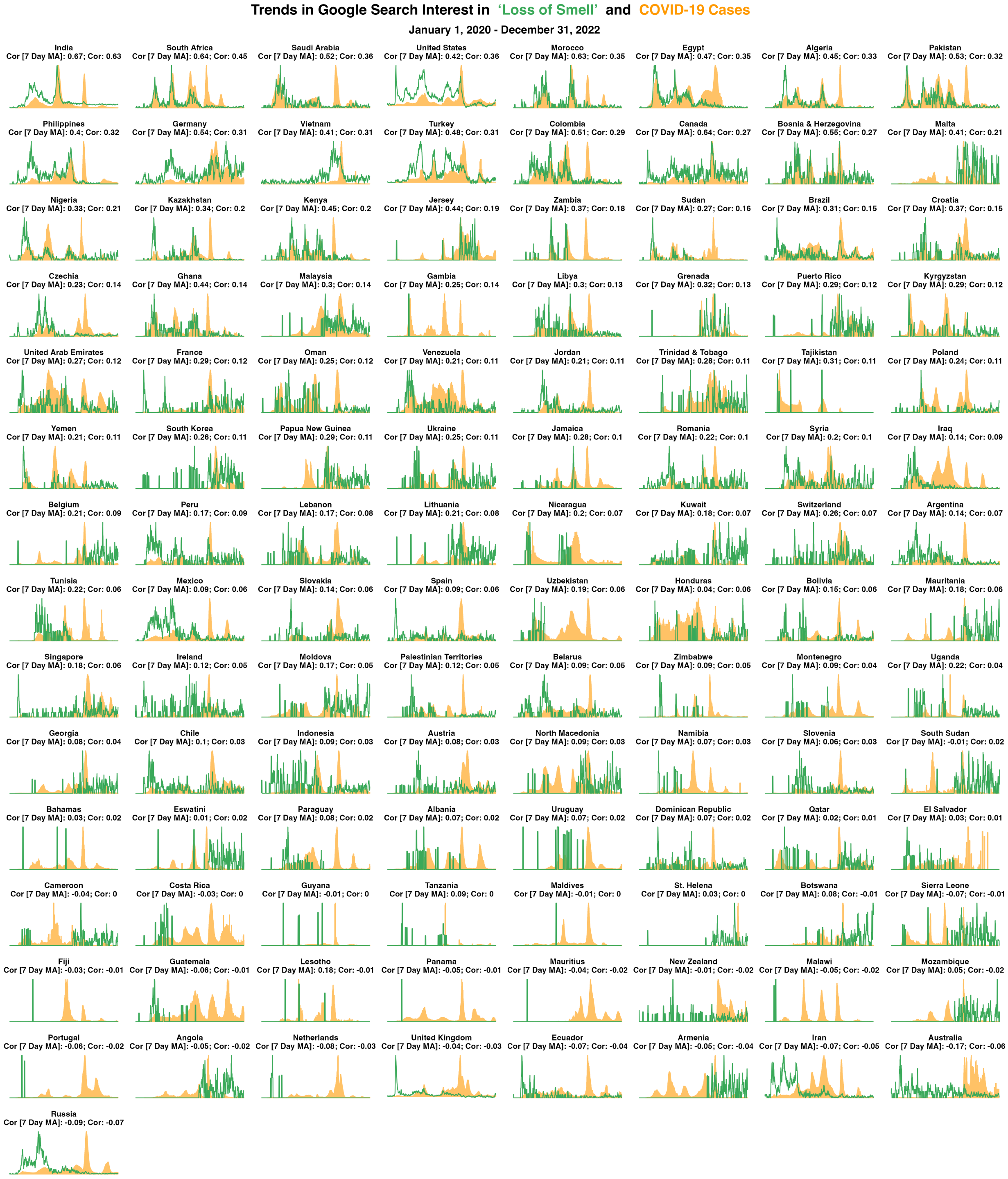

Figure S4: Trends between search interest in "Loss of Smell" and COVID-19 cases for all countries with available data. To show trends more clearly, the seven-day moving average of search interest is shown.

S7 Trends in search interest for "COVID Symptoms" and COVID-19 cases for all countries with available data

Figure S5 shows trends in search interest in "COVID Symptoms" and COVID-19 cases for all countries with available data.

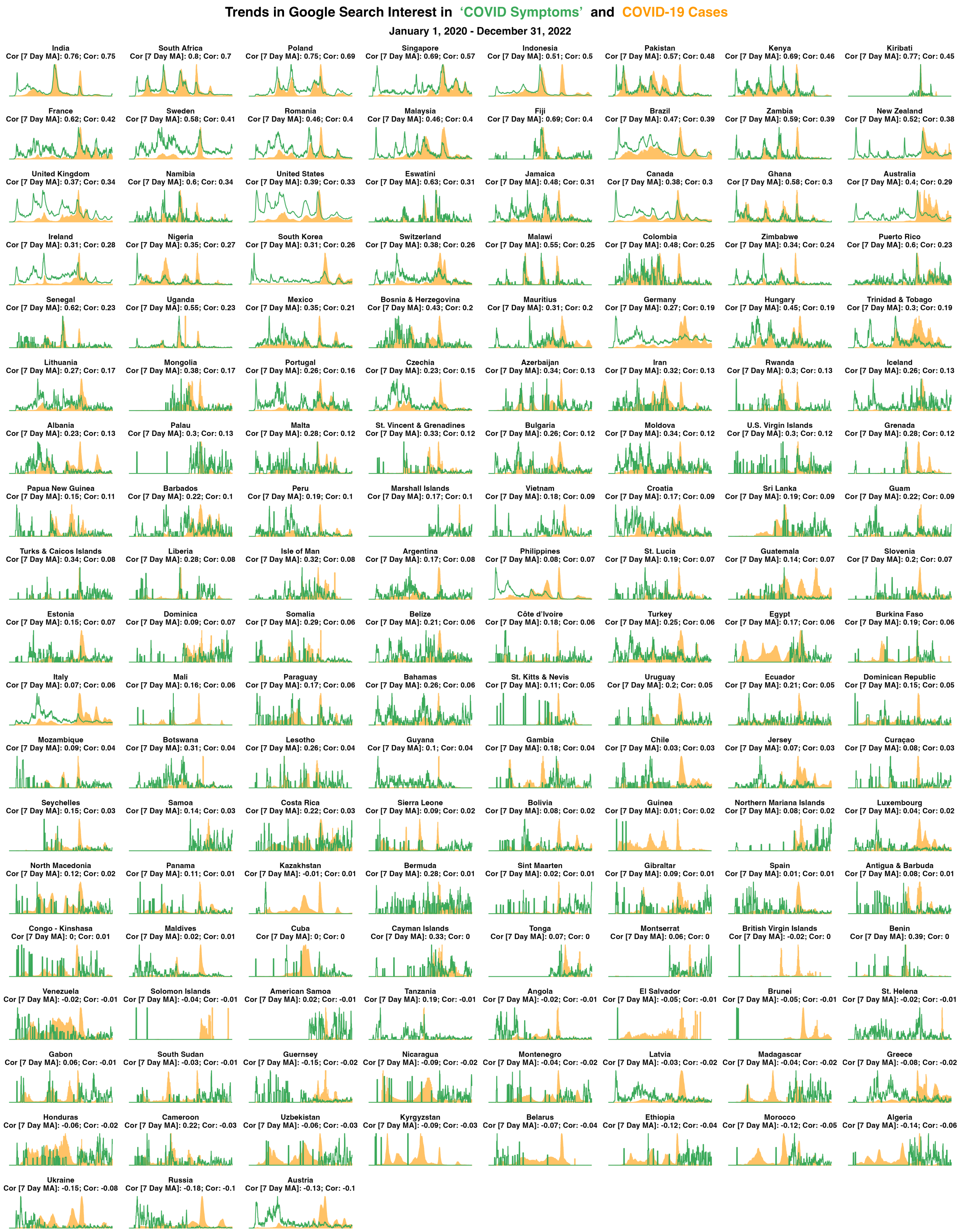

Figure S5: Trends between search interest in "COVID Symptoms" and COVID-19 cases for all countries with available data

S8 Trends in search interest for "Coronavirus" and COVID-19 cases for all countries with available data

Figure S6 shows trends in search interest in "Coronavirus" and COVID-19 cases for all countries with available data.

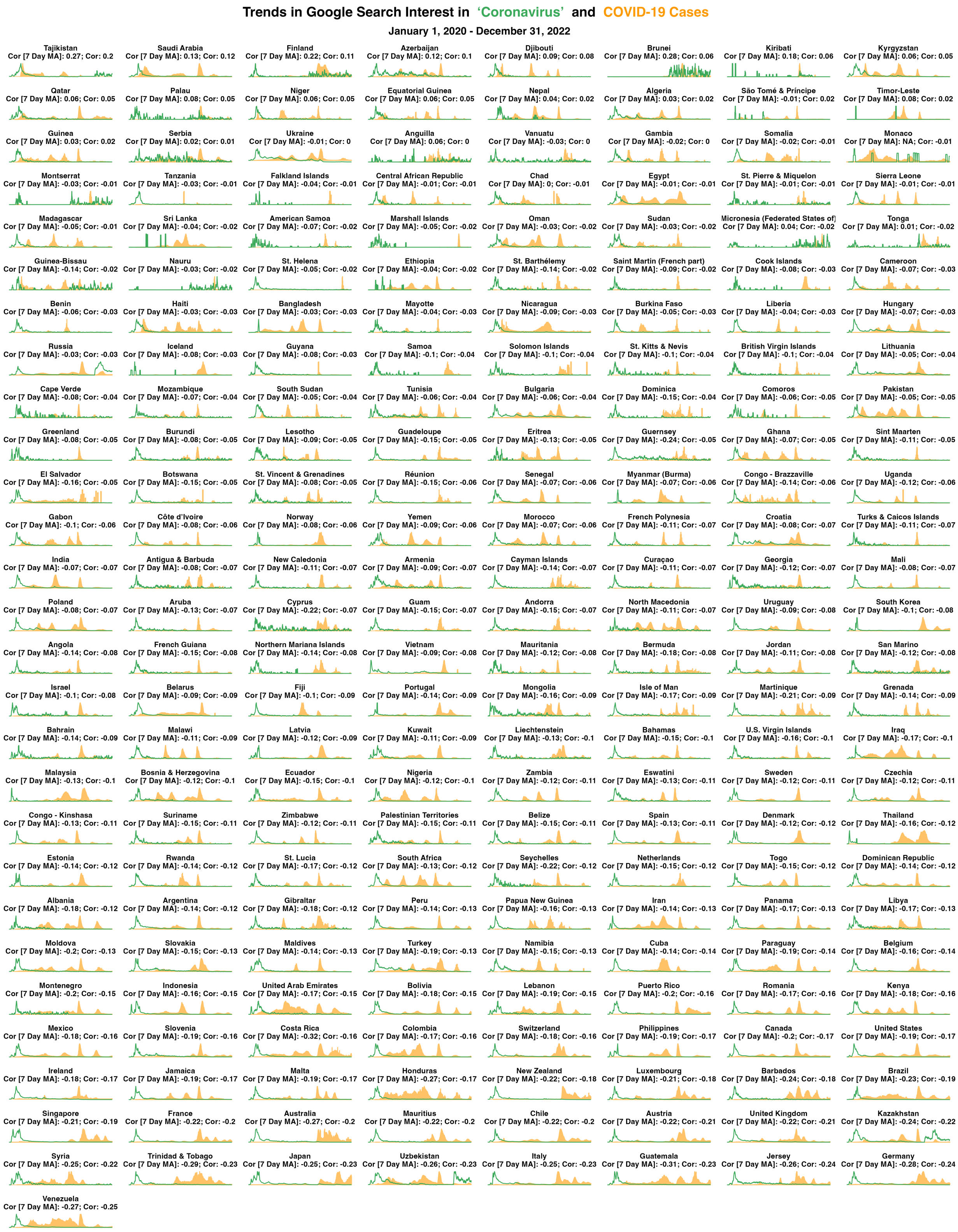

Figure S6: Trends between search interest in "COVID Symptoms" and COVID-19 cases for all countries with available data

S9 Correlation between search interest and reported COVID-19 cases by six months increments

For our main analysis, we examine the correlation between search interest and reported COVID-19 cases annually. As COVID-19 evolved rapidly, annual correlations could mask more granular patterns. Consequently, we also examine correlations at six month increments (see figure S7). The correlation between search interest in “Loss of Smell“ and “Loss of Taste“ with reported COVID-19 cases steadily diminishes over time. The correlation between search interest in “COVID Symptoms“ and reported COVID-19 cases stays relatively consistent over time, except for the second half of 2022 when the median correlation drops.

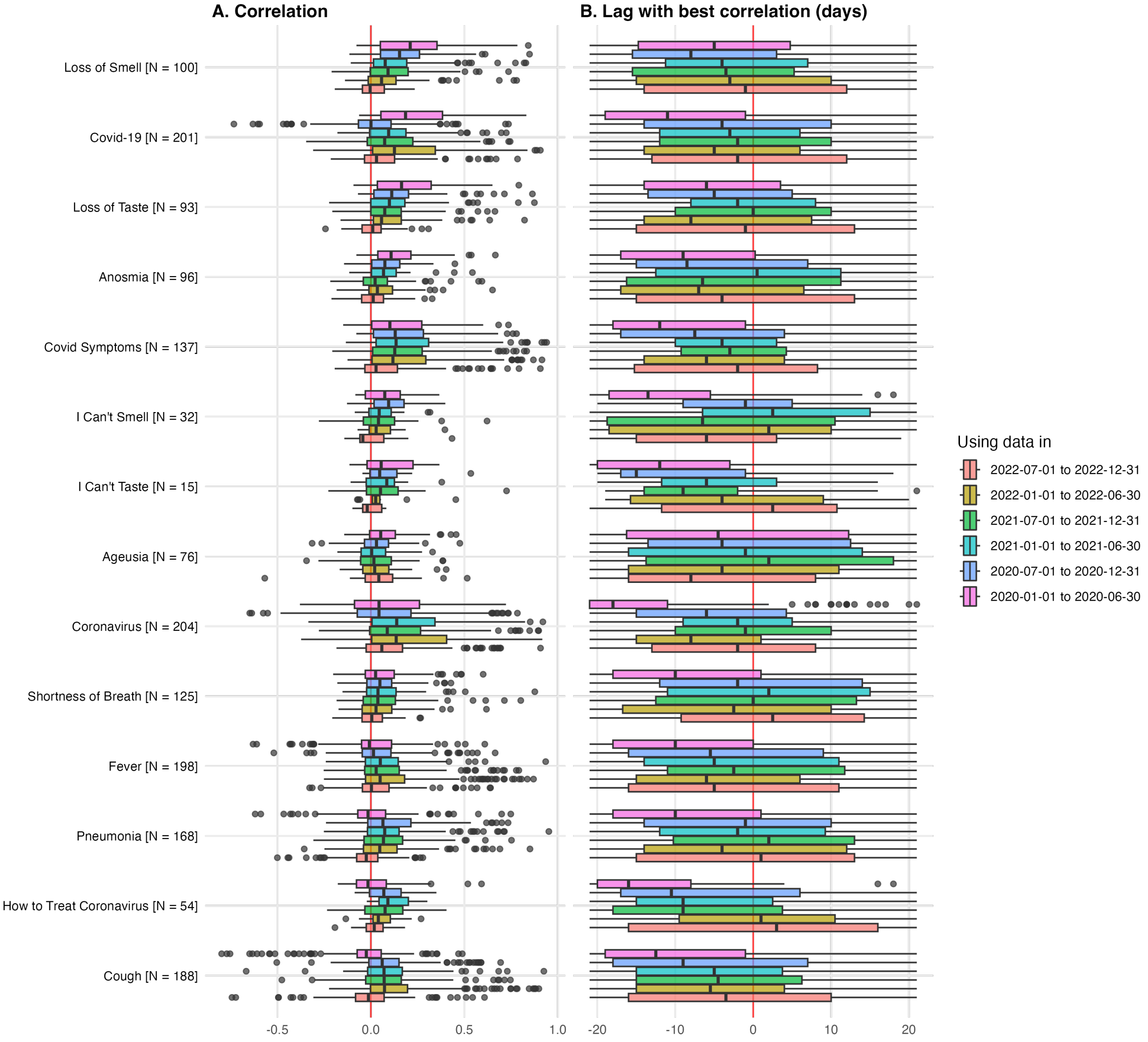

Figure S7: Search interest correlating with and anticipating COVID-19 cases. Panel A shows the correlation between search interest and COVID-19 cases. Panel B shows the lead/lad value of COVID-19 cases that produced the highest correlation with search interest. ‘N’ indicates the number of countries with available data. The boxplots include: center line, median; box limits, upper and lower quartiles; whiskers, 1.5x interquartile range; points beyond whiskers, outliers.

S10 Explaining correlation between search interest and COVID-19 cases: additional results

We estimate OLS models explaining the correlation between “loss of smell” and COVID-19 cases with select covariates. The results show that GDP per capita, percent of the population using internet and the number of mobile cell phone subscribers per 100 are not not associated with the correlation. Here, we test whether these results hold using the correlation between “loss of taste” and COVID-19 cases, and “COVID symptoms” and COVID-19 cases.

Tables S8 and S10 show regression results using the correlation with “loss of taste” and “COVID symptoms” and figures S8 - S11 show scatterplots between all dependent variables and covariates. Results are largely consistent with those that use search interest in “loss of smell” as the dependent variable.

Table S8: Explaining correlation between search interest in "loss of taste" and COVID-19 cases, using data from 2020 and 2021

	Dependent variable:							
	Correlation							
	(1)	(2)	(3)	(4)	(5)	(6)	(7)	(8)
Total COVID-19 Cases, log	0.03***						0.04***	0.04***
	(0.01)						(0.01)	(0.01)
Per Pop. Using Internet		0.0005						−0.001
		(0.001)						(0.001)
Mobile Cell Sub. per 100			0.0002					0.0002
			(0.001)					(0.001)
GDP Per Cap, Log				0.02			−0.01	−0.003
				(0.01)			(0.03)	(0.03)
Low Income					−0.07		0.03	0.03
					(0.07)		(0.11)	(0.11)
Lower Middle Income					−0.03		0.02	0.02
					(0.04)		(0.07)	(0.08)
Upper Middle Income					−0.02		0.01	0.02
					(0.04)		(0.05)	(0.05)
Europe and Central Asia						−0.02	−0.02	−0.02
						(0.05)	(0.05)	(0.05)
Latin America and Caribbean						−0.07	−0.04	−0.04
						(0.05)	(0.05)	(0.05)
Middle East and North Africa						−0.10*	−0.07	−0.06
						(0.05)	(0.05)	(0.06)
North America						0.37***	0.32***	0.32***
						(0.10)	(0.10)	(0.10)
South Asia						0.17**	0.15*	0.14*
						(0.09)	(0.08)	(0.09)
Sub-Saharan Africa						−0.07	−0.001	−0.001
						(0.05)	(0.05)	(0.06)
Constant	−0.35***	0.09*	0.10	−0.03	0.14***	0.16***	−0.27	−0.35
	(0.09)	(0.05)	(0.06)	(0.10)	(0.03)	(0.04)	(0.28)	(0.30)
Observations	105	100	103	102	102	105	102	100
Adjusted R^2	0.20	-0.01	-0.01	0.01	-0.02	0.21	0.32	0.31

*p<0.1; **p<0.05; ***p<0.01

Table S9: Explaining the lead/lag value that produced the highest correlation between search interest in "loss of taste" and COVID-19 cases, using data from 2020 and 2021

	Dependent variable:							
	Best Lag							
	(1)	(2)	(3)	(4)	(5)	(6)	(7)	(8)
Total COVID-19 Cases, log	−0.79						−1.09*	−1.20*
	(0.51)						(0.64)	(0.67)
Per Pop. Using Internet		−0.03						0.04
		(0.05)						(0.11)
Mobile Cell Sub. per 100			0.01					0.002
			(0.04)					(0.04)
GDP Per Cap, Log				−0.20			1.74	0.92
				(0.81)			(2.10)	(2.51)
Low Income					7.13		4.01	4.04
					(4.49)		(8.36)	(8.66)
Lower Middle Income					−1.71		−1.19	−1.21
					(2.54)		(5.83)	(5.93)
Upper Middle Income					3.08		5.53	4.96
					(2.50)		(3.87)	(4.00)
Europe and Central Asia						−2.10	−3.59	−3.38
						(3.48)	(3.55)	(3.68)
Latin America and Caribbean						−7.52**	−9.75**	−9.76**
						(3.68)	(3.81)	(3.90)
Middle East and North Africa						−7.22*	−7.79*	−8.50*
						(4.08)	(4.06)	(4.32)
North America						−1.30	−0.98	−0.44
						(7.52)	(7.55)	(7.89)
South Asia						−5.63	−3.84	−3.47
						(6.39)	(6.41)	(6.68)
Sub-Saharan Africa						3.44	2.45	2.26
						(3.79)	(4.28)	(4.55)
Constant	4.40	−4.61	−8.18*	−4.61	−7.29***	−3.70	−4.58	1.51
	(7.08)	(3.17)	(4.24)	(7.32)	(1.74)	(3.07)	(22.22)	(23.44)
Observations	105	100	103	102	102	105	102	100
Adjusted R^2	0.01	-0.01	-0.01	-0.01	0.03	0.10	0.15	0.13

*p<0.1; **p<0.05; ***p<0.01

Table S10: Explaining correlation between search interest in "COVID symptoms" and COVID-19 cases, using data from 2020 and 2021

	Dependent variable:							
	Correlation							
	(1)	(2)	(3)	(4)	(5)	(6)	(7)	(8)
Total COVID-19 Cases, log	0.03***						0.03***	0.03***
	(0.01)						(0.01)	(0.01)
Per Pop. Using Internet		0.001						−0.004*
		(0.001)						(0.002)
Mobile Cell Sub. per 100			0.0005					−0.0003
			(0.0005)					(0.001)
GDP Per Cap, Log				0.03**			−0.001	0.04
				(0.01)			(0.03)	(0.04)
Low Income					−0.10*		−0.13	−0.19
					(0.05)		(0.13)	(0.15)
Lower Middle Income					−0.07*		−0.10	−0.13
					(0.04)		(0.09)	(0.10)
Upper Middle Income					−0.05		−0.05	−0.04
					(0.04)		(0.06)	(0.06)
Europe and Central Asia						0.04	−0.04	−0.03
						(0.05)	(0.05)	(0.06)
Latin America and Caribbean						−0.02	−0.01	−0.03
						(0.05)	(0.05)	(0.06)
Middle East and North Africa						−0.11	−0.11	−0.06
						(0.09)	(0.09)	(0.09)
North America						0.20*	0.08	0.13
						(0.11)	(0.11)	(0.14)
South Asia						0.21**	0.18*	0.15
						(0.10)	(0.09)	(0.10)
Sub-Saharan Africa						−0.005	0.09	0.05
						(0.05)	(0.06)	(0.07)
Constant	−0.26***	0.11**	0.10*	−0.08	0.20***	0.14***	−0.23	−0.31
	(0.07)	(0.05)	(0.06)	(0.10)	(0.03)	(0.04)	(0.35)	(0.40)
Observations	145	125	135	137	140	145	137	124
Adjusted R^2	0.18	0.01	0.0003	0.03	0.02	0.04	0.21	0.20

*p<0.1; **p<0.05; ***p<0.01

Table S11: Explaining the lead/lag value that produced the highest correlation between search interest in "COVID symptoms" and COVID-19 cases, using data from 2020 and 2021

	Dependent variable:							
	Best Lag							
	(1)	(2)	(3)	(4)	(5)	(6)	(7)	(8)
Total COVID-19 Cases, log	−0.44						−0.37	−0.10
	(0.31)						(0.38)	(0.45)
Per Pop. Using Internet		−0.02						−0.02
		(0.03)						(0.10)
Mobile Cell Sub. per 100			0.01					−0.02
			(0.02)					(0.03)
GDP Per Cap, Log				0.41			2.59	1.05
				(0.57)			(1.79)	(2.22)
Low Income					−0.27		1.98	−3.82
					(2.61)		(7.11)	(7.64)
Lower Middle Income					−1.31		1.71	−2.68
					(2.04)		(5.01)	(5.37)
Upper Middle Income					−2.40		0.59	−1.52
					(1.92)		(3.10)	(3.28)
Europe and Central Asia						−0.35	−0.24	−0.76
						(2.47)	(2.74)	(2.97)
Latin America and Caribbean						−0.19	−0.29	−2.08
						(2.48)	(2.63)	(3.00)
Middle East and North Africa						5.70	6.83	7.27
						(4.51)	(4.65)	(4.77)
North America						4.63	1.30	2.27
						(5.59)	(5.89)	(7.05)
South Asia						−0.70	2.15	1.24
						(4.94)	(5.11)	(5.20)
Sub-Saharan Africa						3.09	6.87**	6.04*
						(2.54)	(3.19)	(3.56)
Constant	−0.83	−5.35**	−7.37***	−10.07*	−5.36***	−7.30***	−27.34	−10.99
	(3.99)	(2.07)	(2.66)	(5.15)	(1.29)	(2.02)	(19.08)	(20.53)
Observations	145	125	135	137	140	145	137	124
Adjusted R^2	0.01	-0.004	-0.01	-0.004	-0.01	-0.003	0.01	-0.0005

*p<0.1; **p<0.05; ***p<0.01

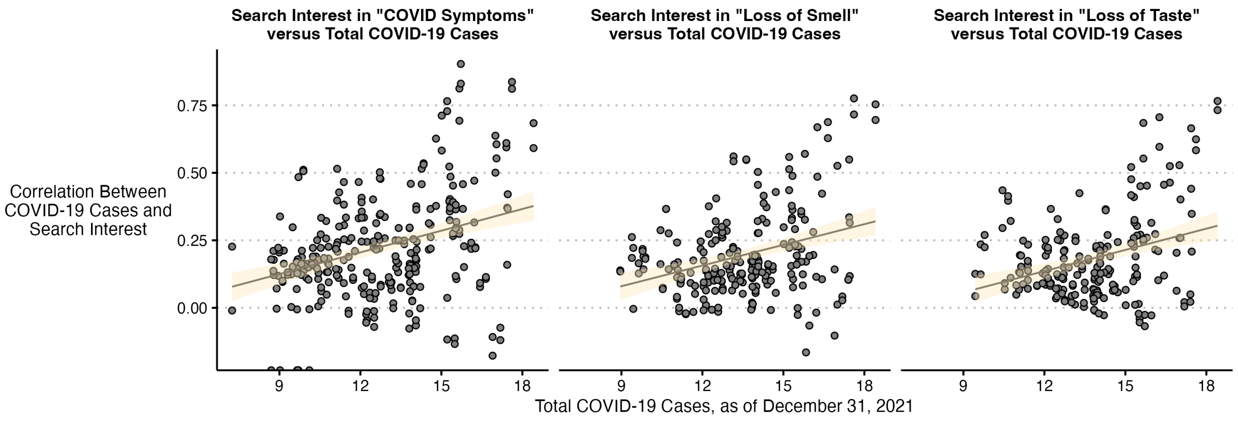

Figure S8: Scatterplots showing association between (1) correlation of search interest and COVID-19 cases and (2) total COVID-19 cases as of December 31, 2021, for search interest in "COVID Symptoms", "Loss of Smell", and "Loss of Taste."

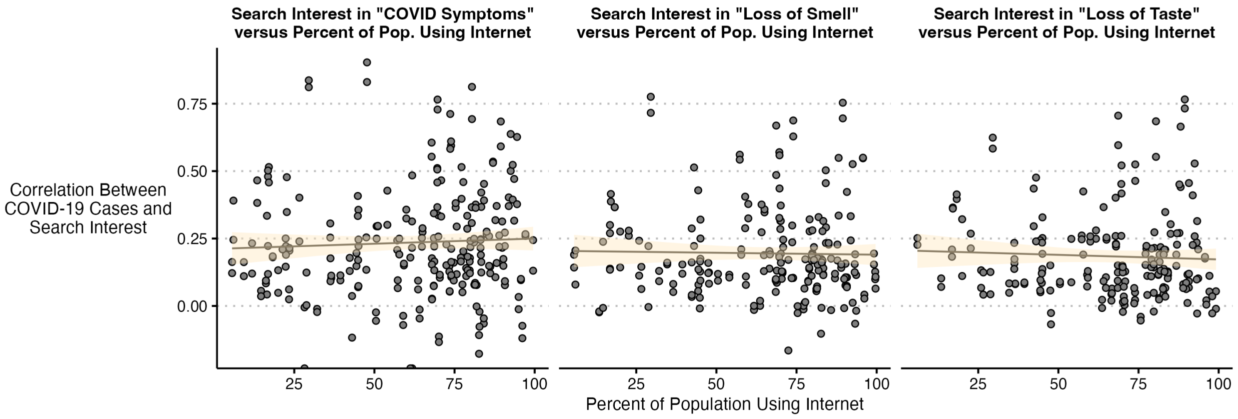

Figure S9: Scatterplots showing association between (1) correlation of search interest and COVID-19 cases and (2) percent of the population using internet, for search interest in "COVID Symptoms", "Loss of Smell", and "Loss of Taste."

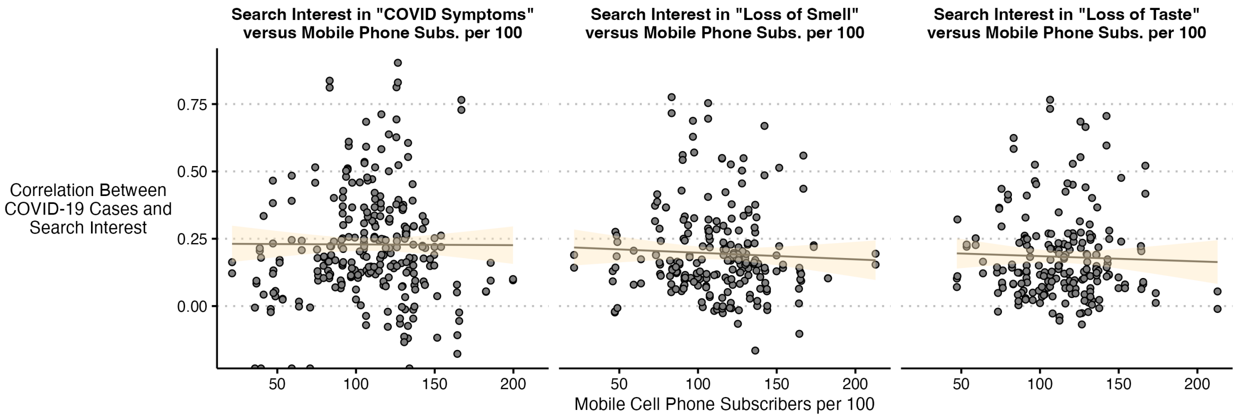

Figure S10: Scatterplots showing association between (1) correlation of search interest and COVID-19 cases and (2) mobile cell phone subscribers per 100, logged, for search interest in "COVID Symptoms", "Loss of Smell", and "Loss of Taste."

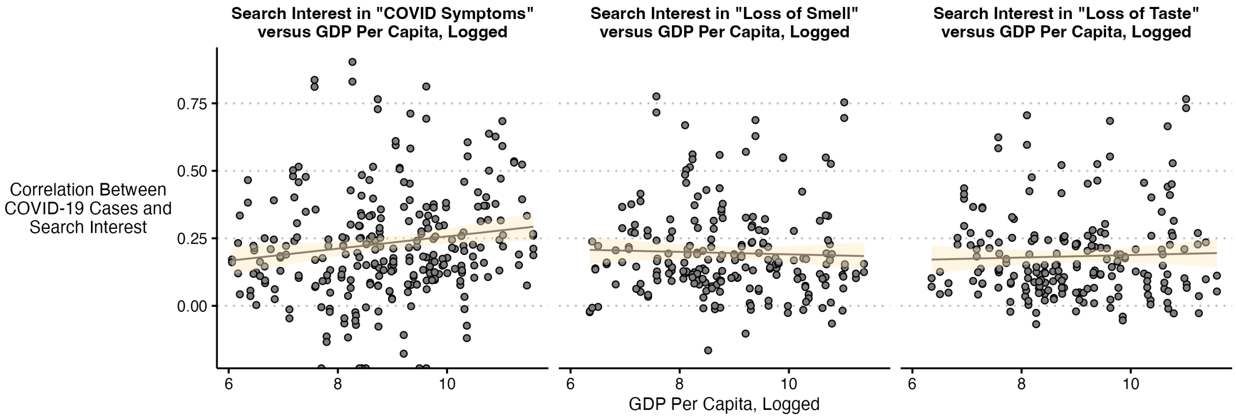

Figure S11: Scatterplots showing association between (1) correlation of search interest and COVID-19 cases and (2) GDP per capita, logged, for search interest in "COVID Symptoms", "Loss of Smell", and "Loss of Taste."

S11 Comparing correlation results using reported COVID-19 cases vs excess mortality

An alternative indicator to measure the burden of COVID-19 is excess mortality [1]. Excess mortality is the additional mortality for a given time and location (e.g., country) compared to expected mortality—such as mortality during similar times in previous years. Excess mortality can help to overcome COVID-19 reporting issues. We use excess mortality data from the WHO; as of this writing, excess mortality is only available from the WHO in 2020 and 2021 [4]. This section compares select results between using reported COVID-19 cases and excess mortality. Excess mortality data is only available monthly from the WHO, so we compare results using monthly reported COVID-19 cases and monthly excess mortality data.

Figure S12 shows that the within-country correlation of reported COVID-19 cases and excess mortality tends to be fairly correlated. 25% of countries have a correlation of 0.49 or higher. Figure S13 shows that search interest across search terms is similarly correlated with reported COVID-19 cases and excess mortality. Tables S12 - S17 shows regressions results explaining the correlation of search interest and COVID-19 cases or excess mortality. The results are largely similar.

We may expect larger differences in results for countries with more limited reporting capacity. In settings where underreporting may be a larger issue—and reported COVID-19 case data to be especially inaccurate—we may expect Google search data to be more strongly associated with a more accurate metrics such as excess mortality. While we don't have a direct metric for testing capacity, we disaggregate results by a country's income level—assuming lower income countires would generally have lower testing capacity. Figure S14 shows results, where results only include the search terms most correlated with reported COVID-19 cases. Contrary to our expectations, the correlation between search interest and COVID-19 appears stronger than the correlation between search interest and excess mortality for lower middle and low income countries.

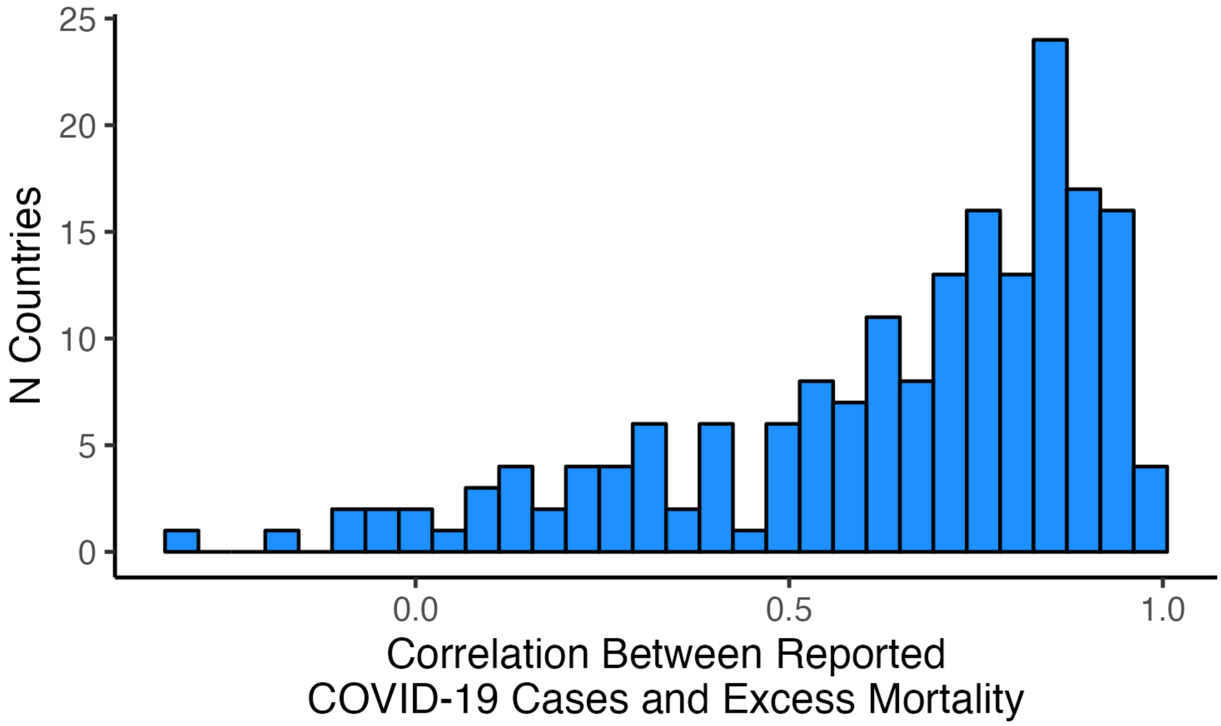

Figure S12: Distribution of within-country correlation between reported COVID-19 cases and excess mortality. Monthly data used from 2020-2021.

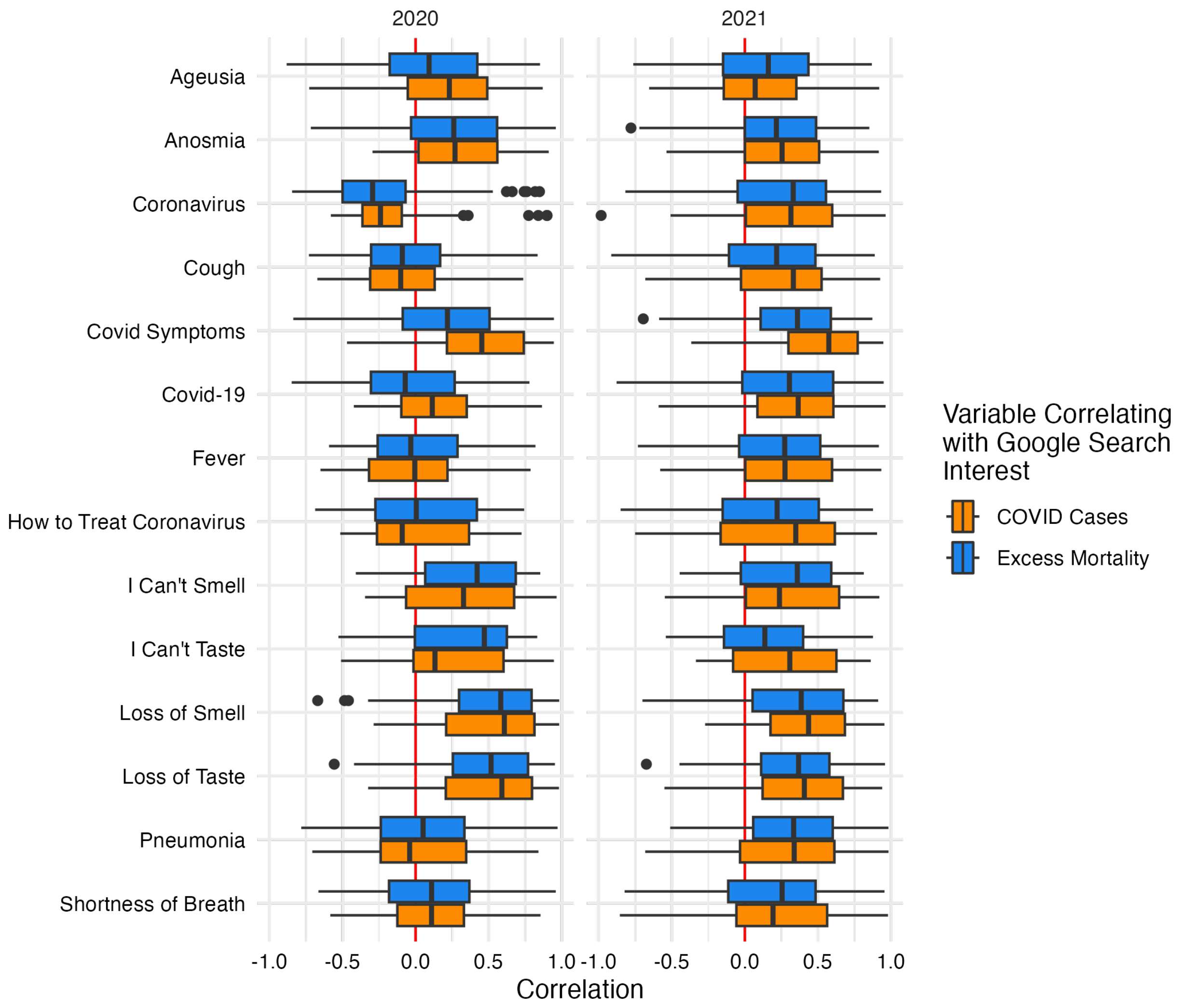

Figure S13: Search interest correlating search interest across search terms and indicators of COVID (COVID cases and excess mortality). Panel A shows the correlation using 2020 data and panel B shows data using 2021 data. The boxplots include: center line, median; box limits, upper and lower quartiles; whiskers, 1.5x interquartile range; points beyond whiskers, outliers.

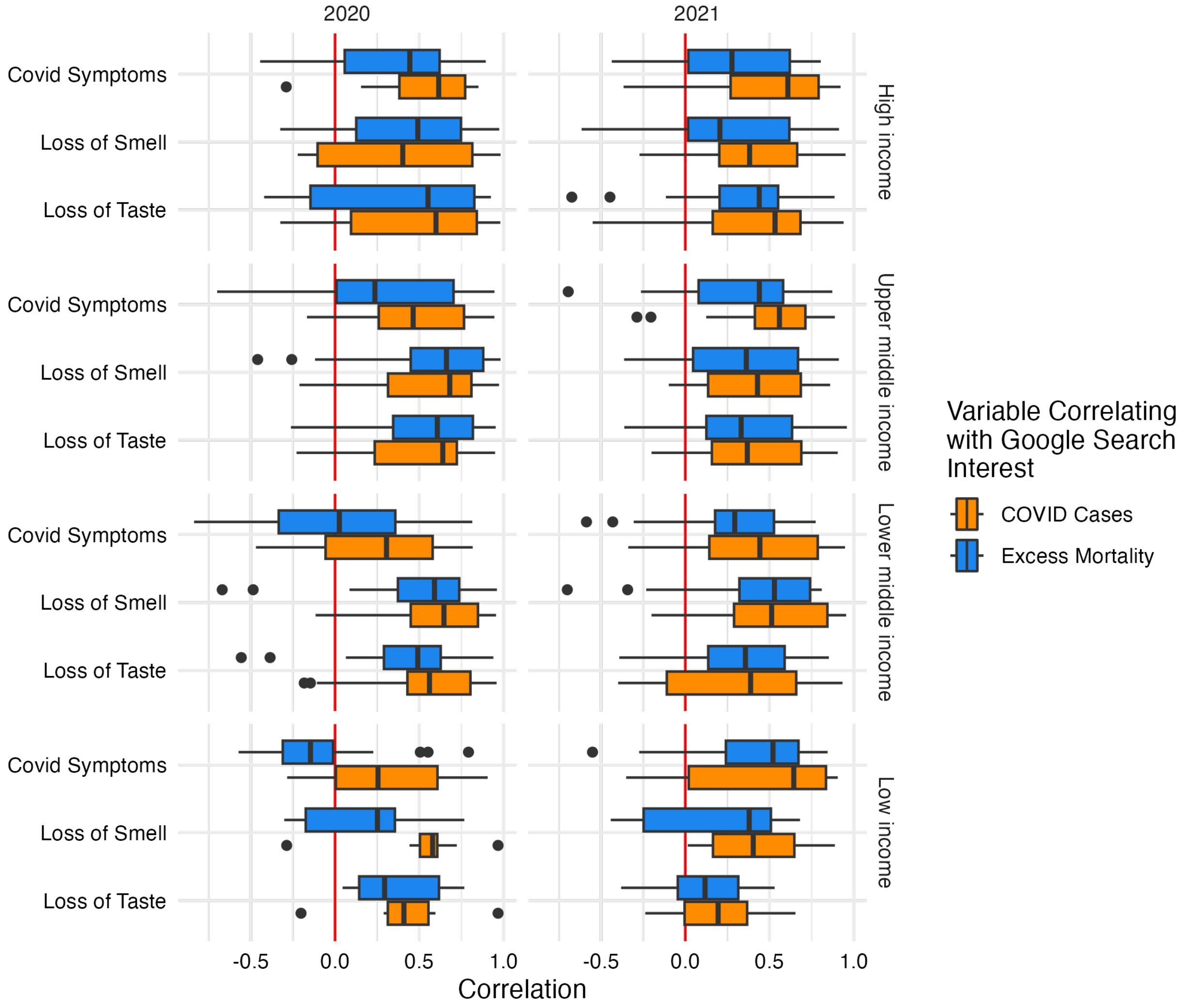

Figure S14: Search interest correlating search interest across search terms and indicators of COVID (COVID cases and excess mortality), by income level and using top Google search interest keywords.

Table S12: Explaining correlation between COVID cases and search interest in Loss of Smell

	Dependent variable:							
	Correlation							
	(1)	(2)	(3)	(4)	(5)	(6)	(7)	(8)
Total COVID-19 Cases, log	0.01						0.02	0.02
	(0.01)						(0.02)	(0.02)
Per Pop. Using Internet		−0.0004						0.002
		(0.001)						(0.003)
Mobile Cell Sub. per 100			0.0004					0.001
			(0.001)					(0.001)
GDP Per Cap, Log				−0.03			−0.10	−0.13*
				(0.02)			(0.06)	(0.08)
Low Income					0.04		−0.07	−0.01
					(0.10)		(0.24)	(0.25)
Lower Middle Income					0.11		−0.01	−0.002
					(0.07)		(0.17)	(0.17)
Upper Middle Income					0.02		−0.04	−0.07
					(0.07)		(0.11)	(0.11)
Europe and Central Asia						0.08	0.08	0.08
						(0.10)	(0.10)	(0.11)
Latin America and Caribbean						0.02	0.01	0.03
						(0.10)	(0.11)	(0.11)
Middle East and North Africa						0.12	0.13	0.10
						(0.11)	(0.11)	(0.12)
North America						0.53**	0.63***	0.67***
						(0.21)	(0.22)	(0.23)
South Asia						0.22	0.10	0.13
						(0.18)	(0.19)	(0.19)
Sub-Saharan Africa						0.005	−0.11	−0.08
						(0.11)	(0.12)	(0.13)
Constant	0.22	0.37***	0.31***	0.62***	0.31***	0.29***	1.00	0.99
	(0.18)	(0.08)	(0.10)	(0.20)	(0.05)	(0.09)	(0.65)	(0.68)
Observations	109	104	108	105	107	109	105	103
Adjusted R^2	-0.004	-0.01	-0.01	0.01	-0.002	0.03	0.08	0.07

Table S13: Explaining correlation between excess mortality and search interest in Loss of Smell

	Dependent variable:							
	Correlation							
	(1)	(2)	(3)	(4)	(5)	(6)	(7)	(8)
Total COVID-19 Cases, log	0.05***						0.06***	0.06***
	(0.01)						(0.02)	(0.02)
Per Pop. Using Internet		0.002*						−0.003
		(0.001)						(0.003)
Mobile Cell Sub. per 100			0.001					0.001
			(0.001)					(0.001)
GDP Per Cap, Log				0.01			−0.09	−0.06
				(0.02)			(0.06)	(0.07)
Low Income					−0.19*		−0.04	−0.06
					(0.11)		(0.23)	(0.24)
Lower Middle Income					0.06		0.08	0.07
					(0.07)		(0.16)	(0.16)
Upper Middle Income					0.07		0.06	0.07
					(0.07)		(0.11)	(0.11)
Europe and Central Asia						0.17*	0.19*	0.20*
						(0.10)	(0.10)	(0.10)
Latin America and Caribbean						0.06	0.09	0.10
						(0.11)	(0.11)	(0.11)
Middle East and North Africa						0.18	0.24**	0.26**
						(0.11)	(0.11)	(0.11)
North America						0.21	0.27	0.29
						(0.22)	(0.21)	(0.22)
South Asia						−0.07	−0.21	−0.24
						(0.18)	(0.18)	(0.19)
Sub-Saharan Africa						−0.15	−0.13	−0.14
						(0.11)	(0.12)	(0.12)
Constant	−0.30*	0.23***	0.21**	0.29	0.35***	0.31***	0.27	0.12
	(0.18)	(0.08)	(0.11)	(0.21)	(0.05)	(0.09)	(0.62)	(0.66)
Observations	109	104	108	105	107	109	105	103
Adjusted R^2	0.11	0.02	0.01	-0.01	0.03	0.13	0.23	0.22

Table S14: Explaining correlation between COVID cases and search interest in Loss of Taste

	Dependent variable:							
	Correlation							
	(1)	(2)	(3)	(4)	(5)	(6)	(7)	(8)
Total COVID-19 Cases, log	0.03*						0.03	0.03*
	(0.01)						(0.02)	(0.02)
Per Pop. Using Internet		−0.0005						−0.002
		(0.001)						(0.003)
Mobile Cell Sub. per 100			−0.001					−0.001
			(0.001)					(0.001)
GDP Per Cap, Log				0.01			0.02	0.07
				(0.02)			(0.06)	(0.07)
Low Income					−0.02		0.10	0.08
					(0.13)		(0.25)	(0.25)
Lower Middle Income					0.0000		0.09	0.08
					(0.07)		(0.17)	(0.17)
Upper Middle Income					0.01		0.07	0.11
					(0.07)		(0.12)	(0.12)
Europe and Central Asia						−0.02	−0.02	−0.04
						(0.10)	(0.11)	(0.11)
Latin America and Caribbean						−0.02	0.002	−0.01
						(0.11)	(0.11)	(0.11)
Middle East and North Africa						−0.12	−0.09	−0.04
						(0.12)	(0.12)	(0.13)
North America						0.49**	0.45*	0.39*
						(0.22)	(0.23)	(0.23)
South Asia						0.25	0.24	0.22
						(0.18)	(0.19)	(0.19)
Sub-Saharan Africa						0.02	0.09	0.11
						(0.11)	(0.13)	(0.13)
Constant	−0.03	0.36***	0.45***	0.26	0.32***	0.33***	−0.29	−0.59
	(0.20)	(0.09)	(0.12)	(0.21)	(0.05)	(0.09)	(0.66)	(0.68)
Observations	102	98	101	100	100	102	100	98
Adjusted R^2	0.02	-0.01	0.001	-0.01	-0.03	0.05	0.03	0.04

Table S15: Explaining correlation between excess mortality and search interest in Loss of Taste

	Dependent variable:							
	Correlation							
	(1)	(2)	(3)	(4)	(5)	(6)	(7)	(8)
Total COVID-19 Cases, log	0.03**						0.04**	0.04*
	(0.01)						(0.02)	(0.02)
Per Pop. Using Internet		−0.0001						−0.01**
		(0.001)						(0.003)
Mobile Cell Sub. per 100			0.0003					0.001
			(0.001)					(0.001)
GDP Per Cap, Log				0.002			−0.03	0.06
				(0.02)			(0.06)	(0.07)
Low Income					−0.08		−0.02	−0.07
					(0.13)		(0.26)	(0.25)
Lower Middle Income					0.02		0.05	0.04
					(0.07)		(0.18)	(0.17)
Upper Middle Income					0.11		0.13	0.18
					(0.07)		(0.12)	(0.12)
Europe and Central Asia						0.16	0.16	0.18*
						(0.11)	(0.11)	(0.11)
Latin America and Caribbean						0.10	0.09	0.08
						(0.11)	(0.12)	(0.12)
Middle East and North Africa						0.13	0.15	0.25*
						(0.13)	(0.13)	(0.13)
North America						0.29	0.30	0.34
						(0.23)	(0.23)	(0.23)
South Asia						0.17	0.10	0.02
						(0.19)	(0.20)	(0.20)
Sub-Saharan Africa						0.06	0.11	0.06
						(0.12)	(0.13)	(0.13)
Constant	−0.07	0.37***	0.33***	0.35*	0.33***	0.25***	−0.08	−0.56
	(0.20)	(0.09)	(0.12)	(0.21)	(0.05)	(0.09)	(0.68)	(0.69)
Observations	102	98	101	100	100	102	100	98
Adjusted R^2	0.04	-0.01	-0.01	-0.01	0.01	-0.02	0.01	0.06

Table S16: Explaining correlation between COVID cases and search interest in Loss of Taste

	Dependent variable:							
	Correlation							
	(1)	(2)	(3)	(4)	(5)	(6)	(7)	(8)
Total COVID-19 Cases, log	0.01						0.02	0.02
	(0.01)						(0.01)	(0.02)
Per Pop. Using Internet		−0.0002						−0.01**
		(0.001)						(0.003)
Mobile Cell Sub. per 100			0.0003					−0.001
			(0.001)					(0.001)
GDP Per Cap, Log				0.02			−0.01	0.06
				(0.02)			(0.07)	(0.08)
Low Income					−0.06		−0.31	−0.51*
					(0.09)		(0.25)	(0.26)
Lower Middle Income					−0.12		−0.25	−0.35*
					(0.07)		(0.18)	(0.18)
Upper Middle Income					0.01		−0.05	−0.03
					(0.07)		(0.11)	(0.11)
Europe and Central Asia						0.06	−0.05	−0.04
						(0.09)	(0.10)	(0.10)
Latin America and Caribbean						0.11	0.07	0.04
						(0.09)	(0.10)	(0.10)
Middle East and North Africa						−0.13	−0.05	0.04
						(0.16)	(0.16)	(0.16)
North America						0.22	0.05	0.03
						(0.24)	(0.24)	(0.24)
South Asia						0.27	0.32*	0.24
						(0.17)	(0.17)	(0.18)
Sub-Saharan Africa						0.14	0.29**	0.21*
						(0.09)	(0.11)	(0.12)
Constant	0.25	0.40***	0.35***	0.24	0.42***	0.29***	0.28	0.26
	(0.16)	(0.07)	(0.09)	(0.19)	(0.05)	(0.08)	(0.69)	(0.70)
Observations	129	124	128	126	127	129	126	123
Adjusted R^2	-0.002	-0.01	-0.01	-0.003	0.01	0.01	0.06	0.10

Table S17: Explaining correlation between excess mortality and search interest in Loss of Taste

	Dependent variable:							
	Correlation							
	(1)	(2)	(3)	(4)	(5)	(6)	(7)	(8)
Total COVID-19 Cases, log	0.03***						0.03**	0.03*
	(0.01)						(0.01)	(0.02)
Per Pop. Using Internet		0.002						−0.01
		(0.001)						(0.003)
Mobile Cell Sub. per 100			0.001					−0.0004
			(0.001)					(0.001)
GDP Per Cap, Log				0.04*			−0.05	0.02
				(0.02)			(0.07)	(0.08)
Low Income					−0.15		−0.32	−0.42
					(0.09)		(0.26)	(0.27)
Lower Middle Income					−0.18**		−0.27	−0.33*
					(0.07)		(0.18)	(0.19)
Upper Middle Income					0.01		−0.04	−0.01
					(0.07)		(0.11)	(0.12)
Europe and Central Asia						0.26***	0.13	0.13
						(0.09)	(0.10)	(0.10)
Latin America and Caribbean						0.23**	0.20**	0.16
						(0.10)	(0.10)	(0.11)
Middle East and North Africa						0.12	0.18	0.23
						(0.16)	(0.16)	(0.17)
North America						0.34	0.16	0.14
						(0.24)	(0.24)	(0.25)
South Asia						0.22	0.22	0.15
						(0.18)	(0.18)	(0.18)
Sub-Saharan Africa						0.16	0.26**	0.19
						(0.10)	(0.11)	(0.12)
Constant	−0.18	0.16**	0.14	−0.08	0.32***	0.07	0.18	0.11
	(0.16)	(0.07)	(0.10)	(0.19)	(0.05)	(0.08)	(0.69)	(0.72)
Observations	129	124	128	126	127	129	126	123
Adjusted R^2	0.05	0.01	0.003	0.02	0.05	0.02	0.08	0.07

S12 Association of containment policies with search interest: event study results

Our primary approach to estimate the association of containment policies with search interest relies on a difference-in-difference approach. As a robustness check, we also estimate results relying on an event-study approach; figure S12 shows results using data 90 days before and after the first containment policy for each country, where results are largely consistent with difference-in-difference results.

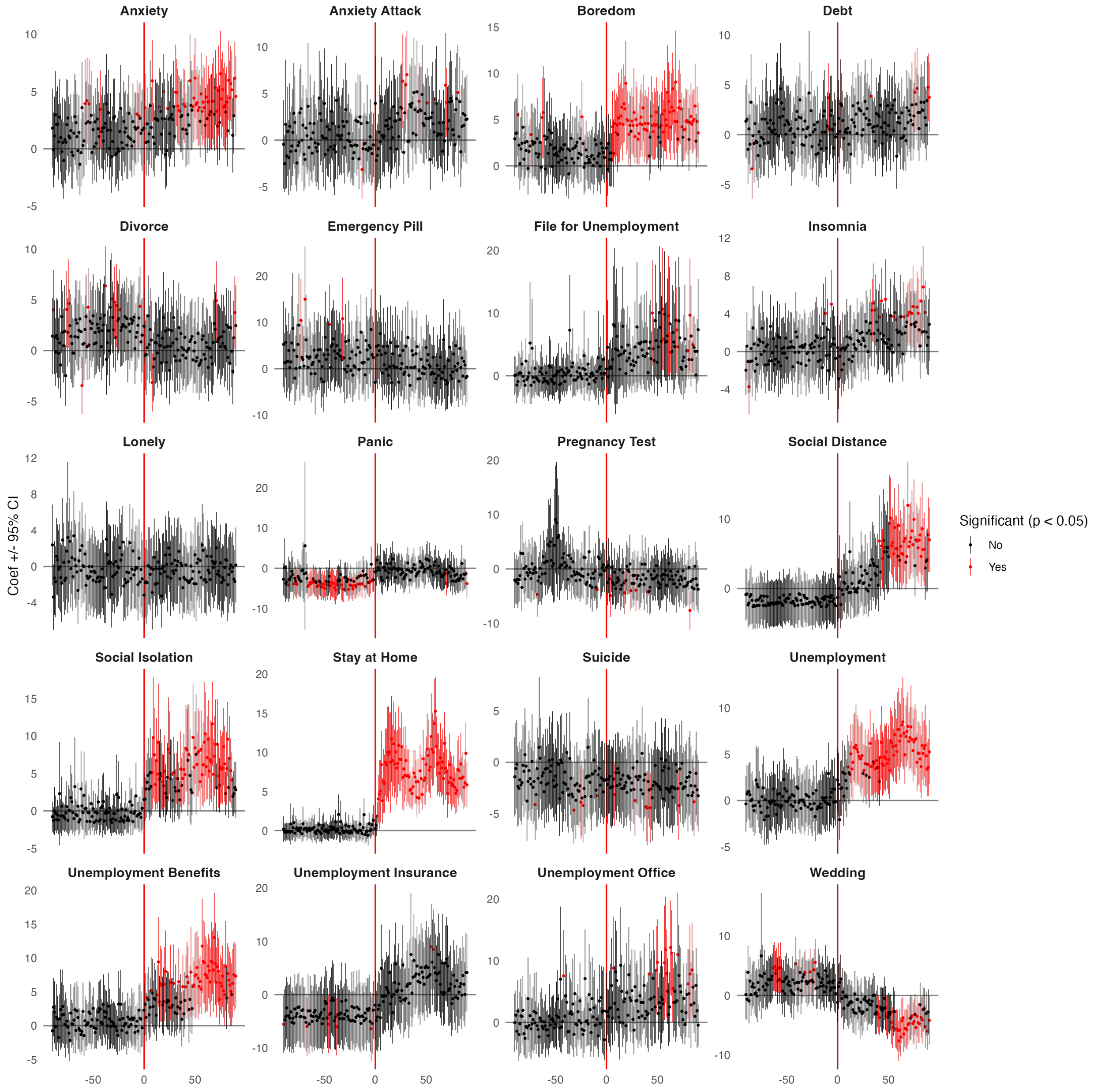

Figure S15: Event study examining the association of containment policies with search interest. 95% confidence intervals shown for each coefficient.

S13 Association of Containment Policies with Search Interest: Sensitivity Analysis Across Different Day Thresholds

To estimate the association of containment policies with search interest, our primary model relies on using days three months (90 days) before and after the date of the first containment policy. This section shows results using 30, 60, 120 and 180 day thresholds.

S13.1 30 day threshold

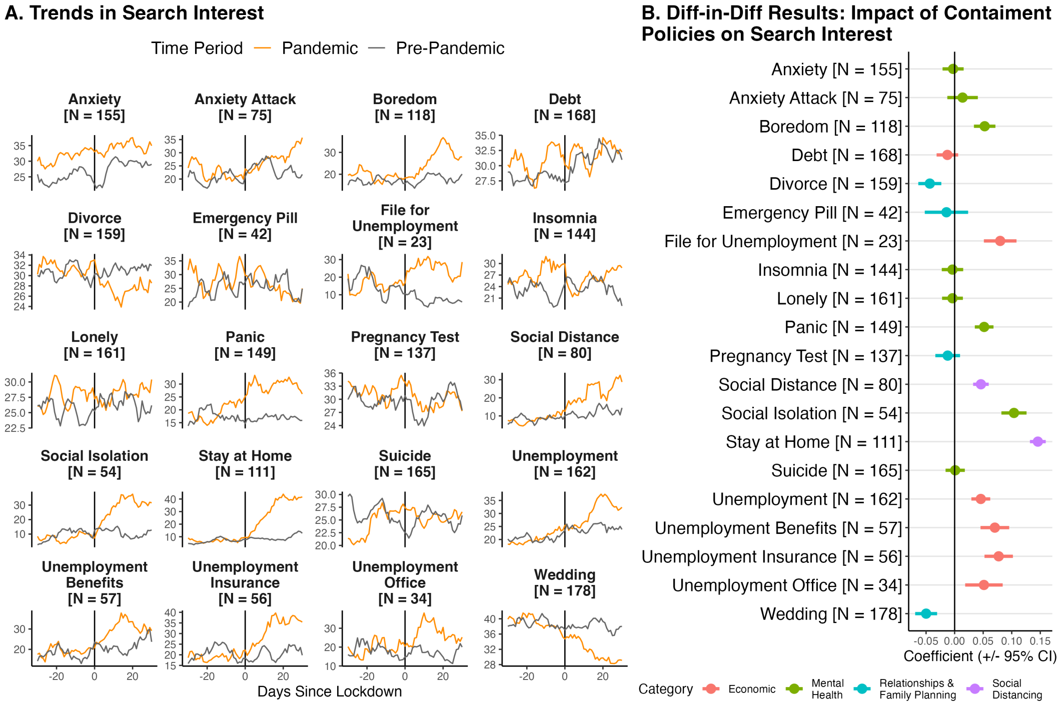

Figure S16: Association of COVID-19 policies with search interest: results pooling all countries. Point estimates and 95% confidence intervals are shown. To more clearly show trends, the seven day moving average of search interest is shown in panel A. 'N' indicates the number of countries with available data.

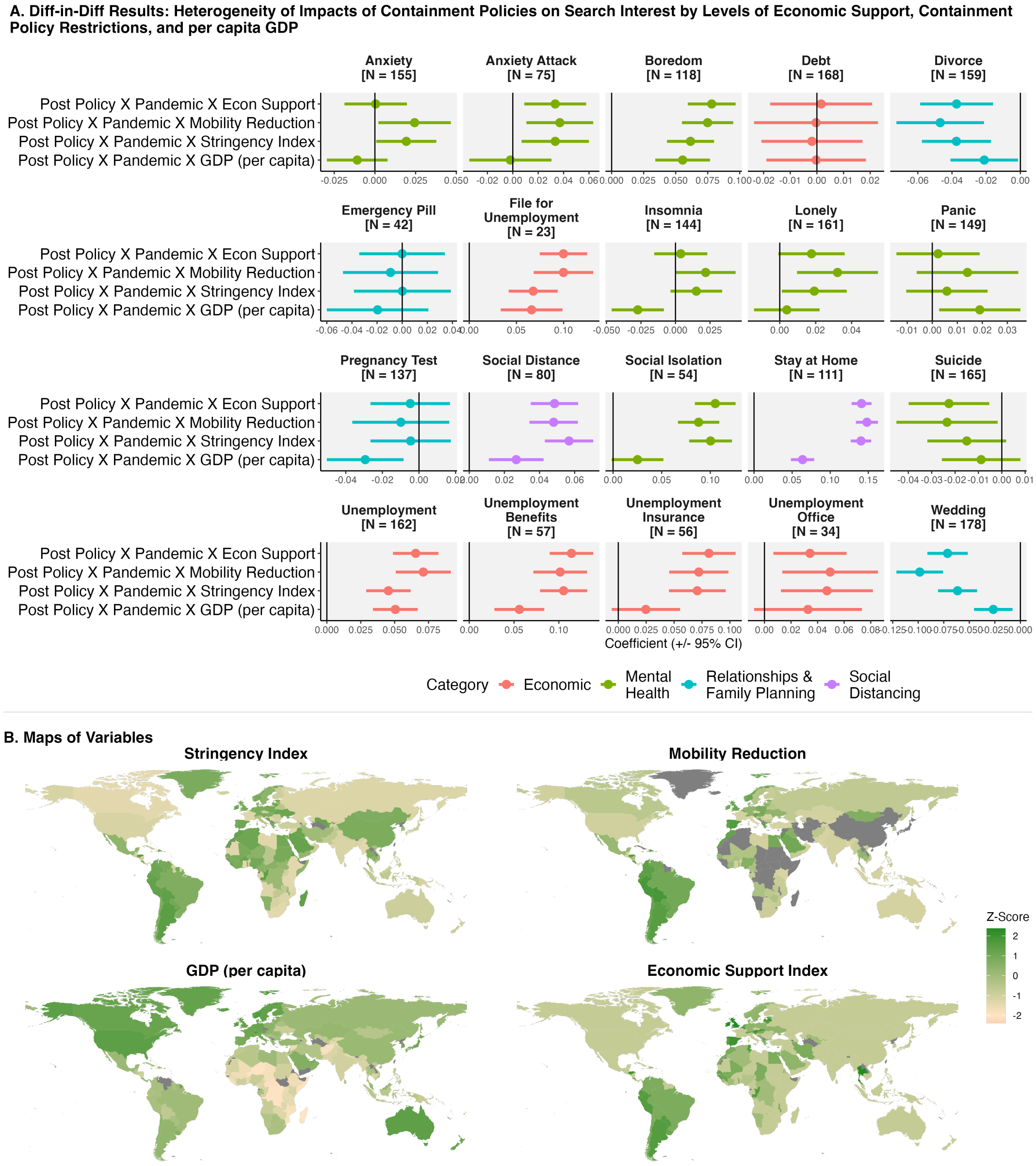

Figure S17: Association of COVID-19 policies on search interest: difference-in-differences results that explore heterogeneity of results across containment policy restrictiveness, economic support, and GDP per capita. Each coefficient comes from a separate regression. The stringency index comes from the University of Oxford COVID-19 Government Response tracker, a composite measure of the restrictiveness of policy measures. Mobility reduction comes from Google COVID-19 Community Mobility Reports, which measure the percent change in mobility relative to pre-pandemic levels. Per capita GDP comes from the World Bank's World Development Indicators; we use log per capita GDP. The Economic Support index from the Oxford COVID-19 Government Response tracker, which measures the extent of economic support across metrics such as income support and debt relief. We standardize all variables into z-scores—having a mean of zero and standard deviation of one. 'N' indicates the number of countries with available data. Maps produced using R, version 4.2.2 (`https://www.r-project.org/`); data for country boundaries come from Natural Earth (`https://www.naturalearthdata.com/`).

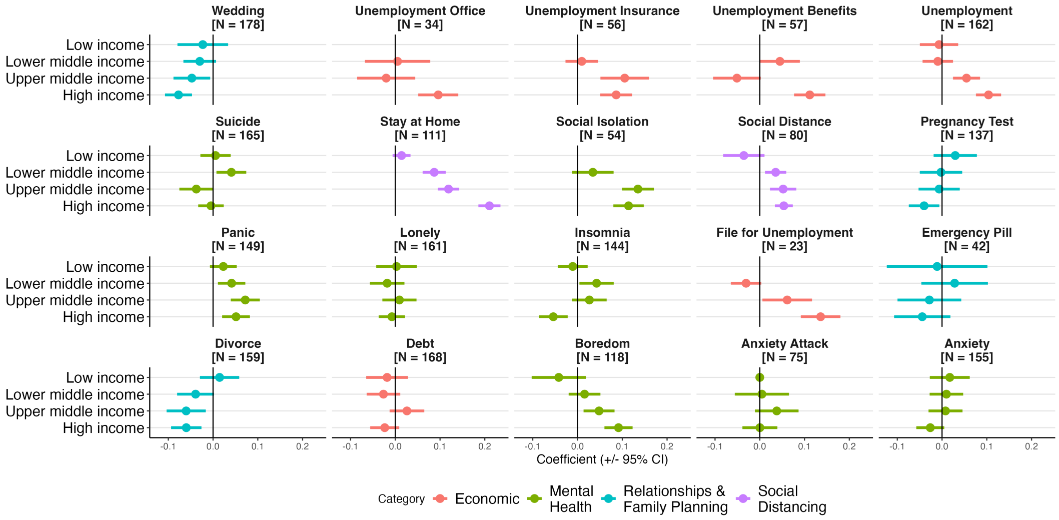

Figure S18: Association of COVID-19 policies with search interest: difference-in-difference results pooling countries by income level. Point estimates and 95% confidence intervals are shown. 'N' indicates the number of countries with available data.

S13.2 60 day threshold

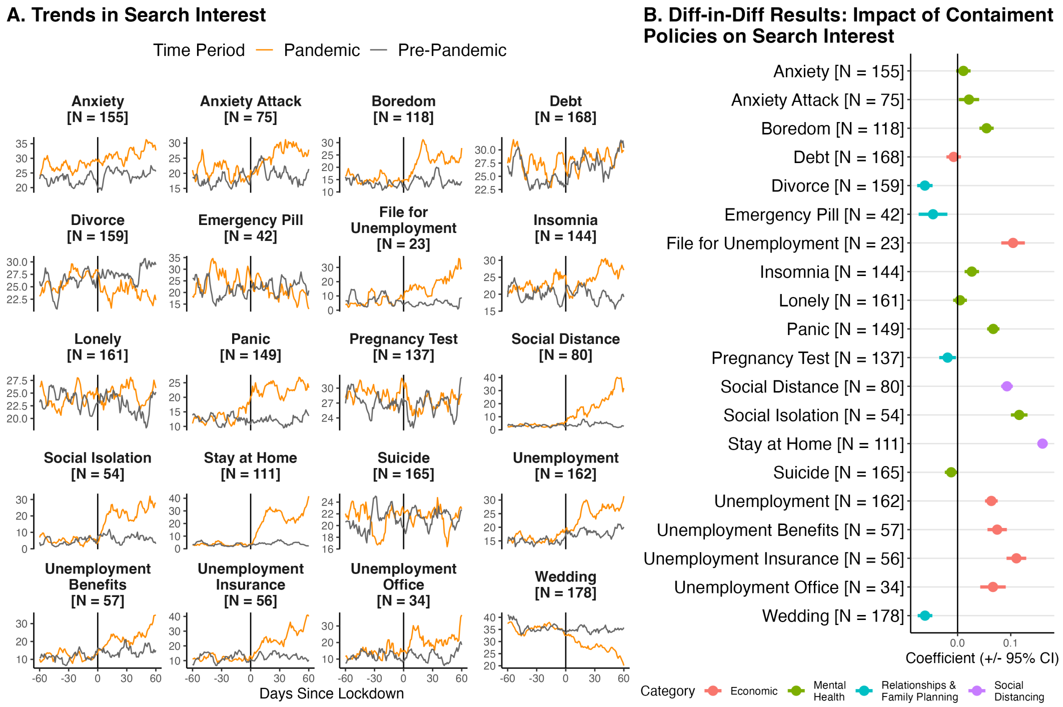

Figure S19: Association of COVID-19 policies with search interest: results pooling all countries. Point estimates and 95% confidence intervals are shown. To more clearly show trends, the seven day moving average of search interest is shown in panel A. ‘N’ indicates the number of countries with available data.

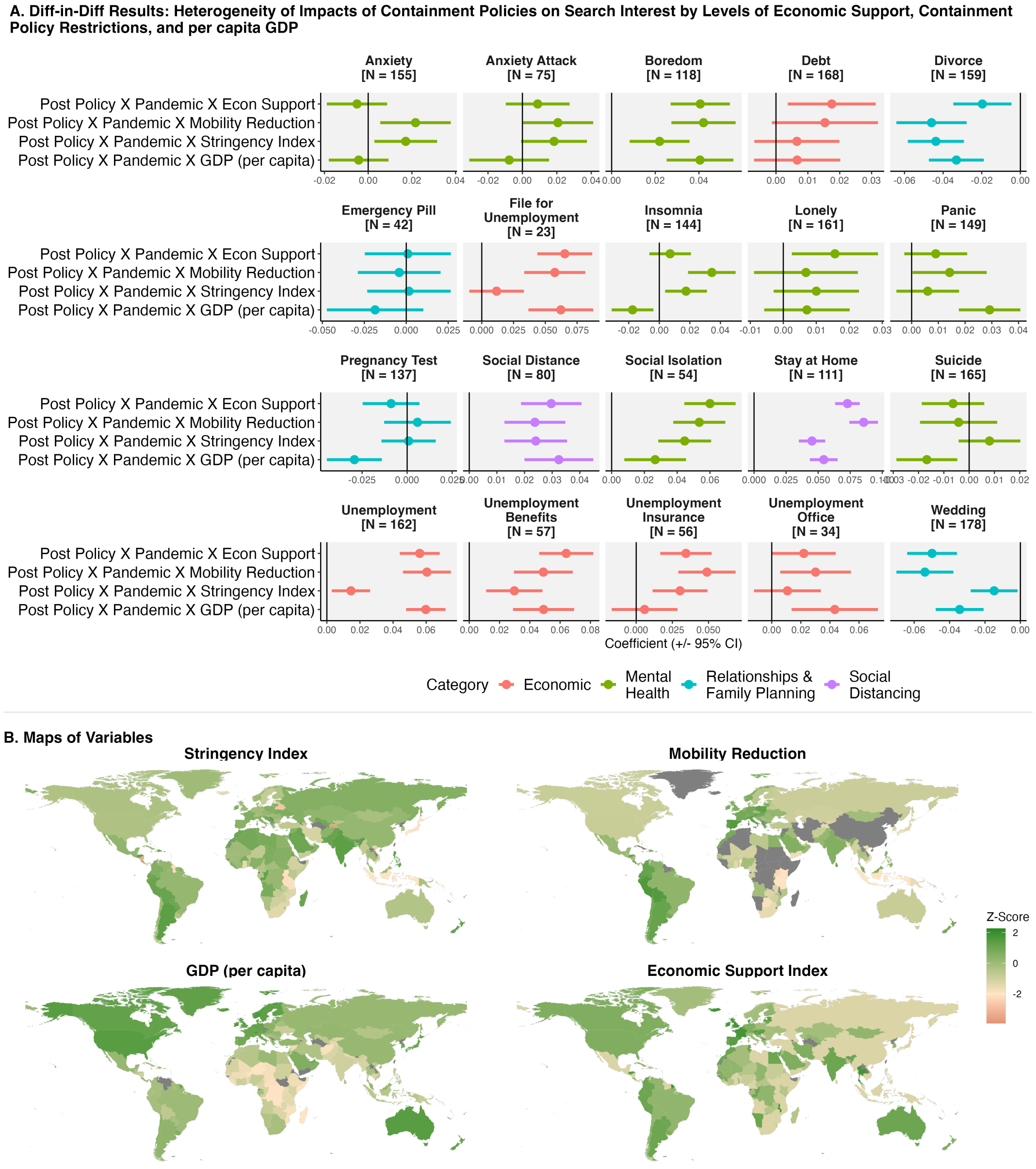

Figure S20: Association of COVID-19 policies with search interest: difference-in-differences results that explore heterogeneity of results across containment policy restrictiveness, economic support, and GDP per capita. Each coefficient comes from a separate regression. The stringency index comes from the University of Oxford COVID-19 Government Response tracker, a composite measure of the restrictiveness of policy measures. Mobility reduction comes from Google COVID-19 Community Mobility Reports, which measure the percent change in mobility relative to pre-pandemic levels. Per capita GDP comes from the World Bank's World Development Indicators; we use log per capita GDP. The Economic Support index from the Oxford COVID-19 Government Response tracker, which measures the extent of economic support across metrics such as income support and debt relief. We standardize all variables into z-scores—having a mean of zero and standard deviation of one. 'N' indicates the number of countries with available data. Maps produced using R, version 4.2.2 (`https://www.r-project.org/`); data for country boundaries come from Natural Earth (`https://www.naturalearthdata.com/`).

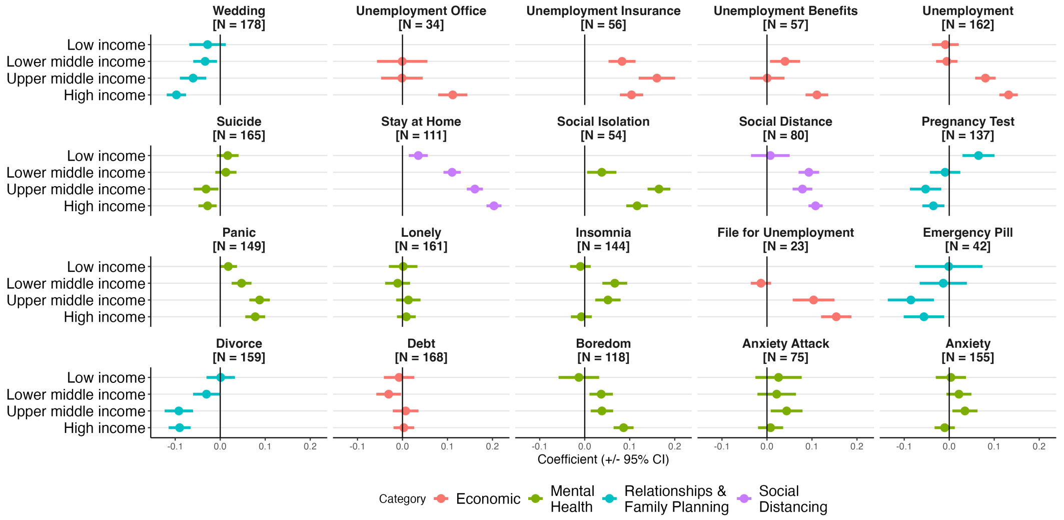

Figure S21: Association of COVID-19 policies with search interest: difference-in-difference results pooling countries by income level. Point estimates and 95% confidence intervals are shown. 'N' indicates the number of countries with available data.

S13.3 120 day threshold

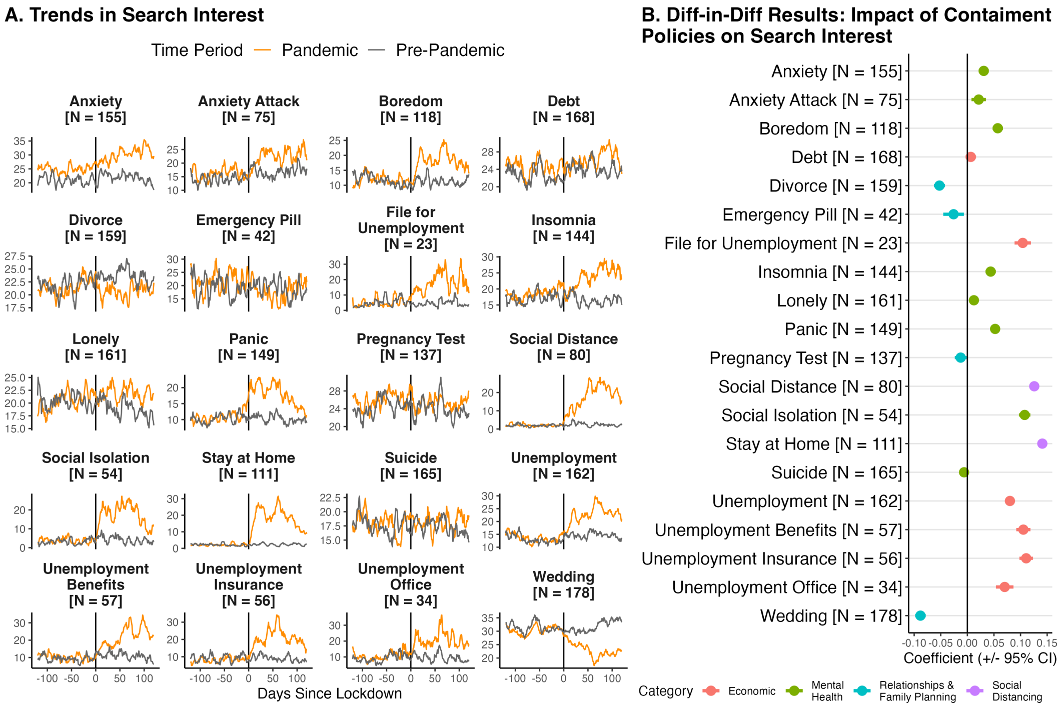

Figure S22: Association of COVID-19 policies with search interest: results pooling all countries. Point estimates and 95% confidence intervals are shown. To more clearly show trends, the seven day moving average of search interest is shown in panel A. 'N' indicates the number of countries with available data.

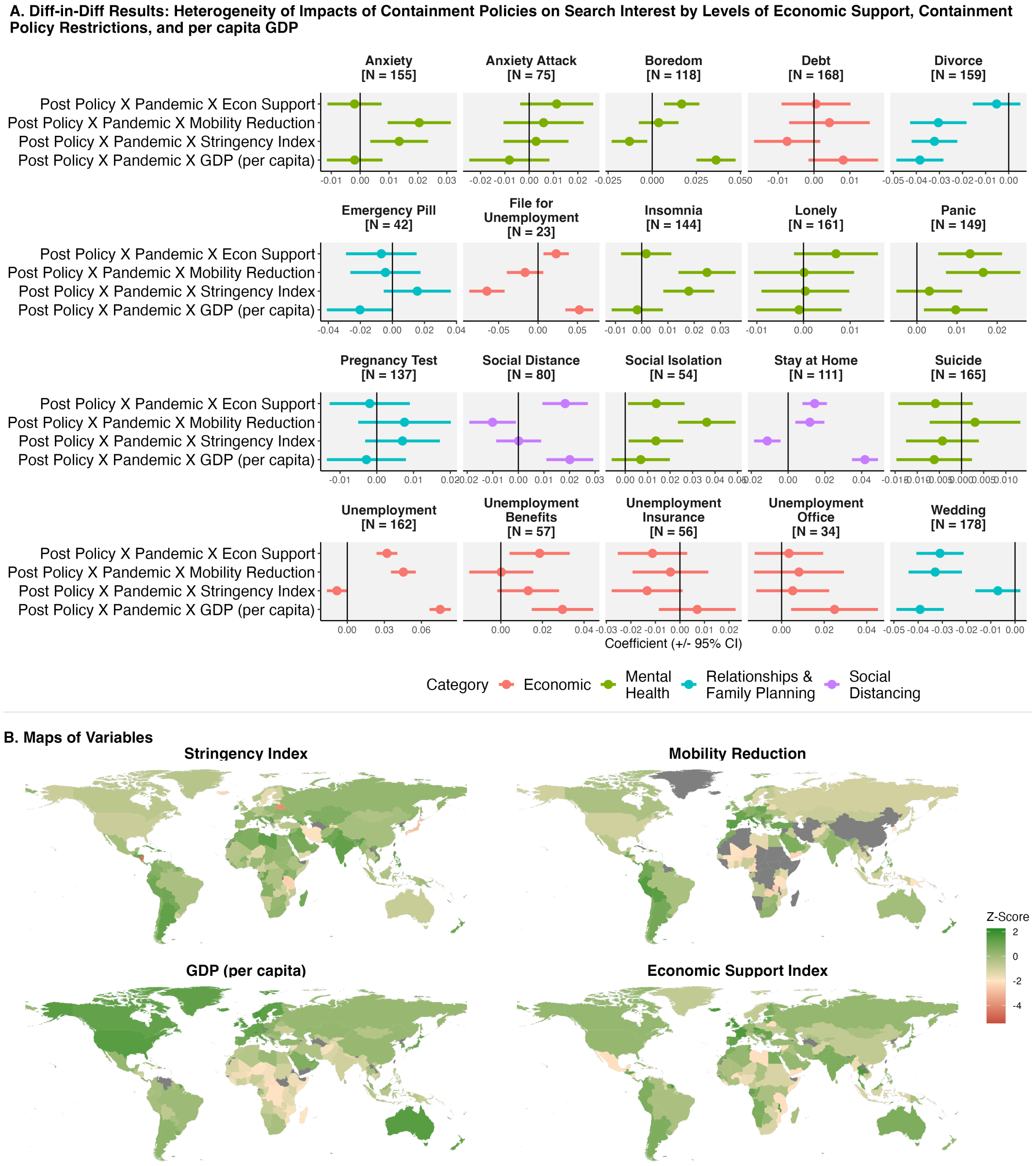

Figure S23: Association of COVID-19 policies with search interest: difference-in-differences results that explore heterogeneity of results across containment policy restrictiveness, economic support, and GDP per capita. Each coefficient comes from a separate regression. The stringency index comes from the University of Oxford COVID-19 Government Response tracker, a composite measure of the restrictiveness of policy measures. Mobility reduction comes from Google COVID-19 Community Mobility Reports, which measure the percent change in mobility relative to pre-pandemic levels. Per capita GDP comes from the World Bank's World Development Indicators; we use log per capita GDP. The Economic Support index from the Oxford COVID-19 Government Response tracker, which measures the extent of economic support across metrics such as income support and debt relief. We standardize all variables into z-scores—having a mean of zero and standard deviation of one. 'N' indicates the number of countries with available data. Maps produced using R, version 4.2.2 (`https://www.r-project.org/`); data for country boundaries come from Natural Earth (`https://www.naturalearthdata.com/`).

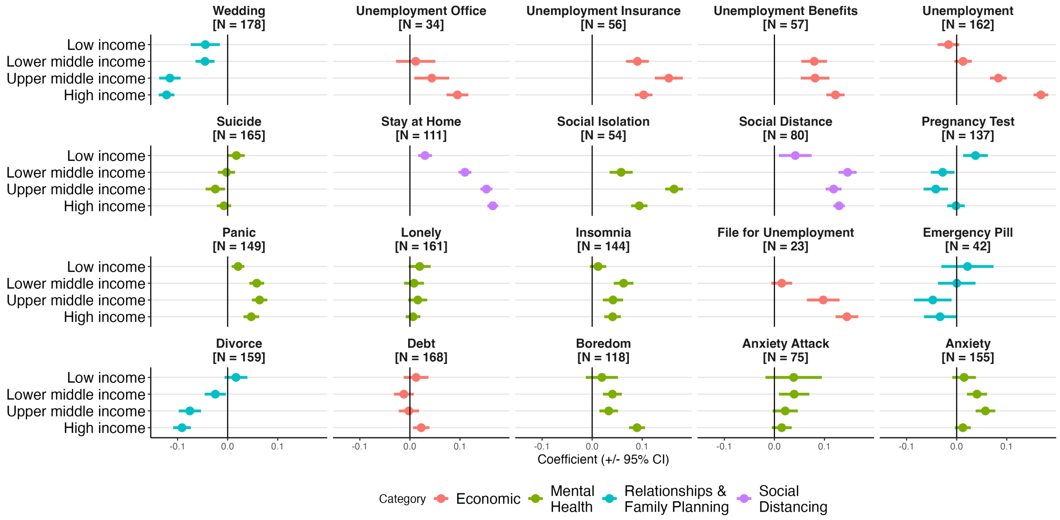

Figure S24: Association of COVID-19 policies with search interest: difference-in-difference results pooling countries by income level. Point estimates and 95% confidence intervals are shown. 'N' indicates the number of countries with available data.

S13.4 180 day threshold

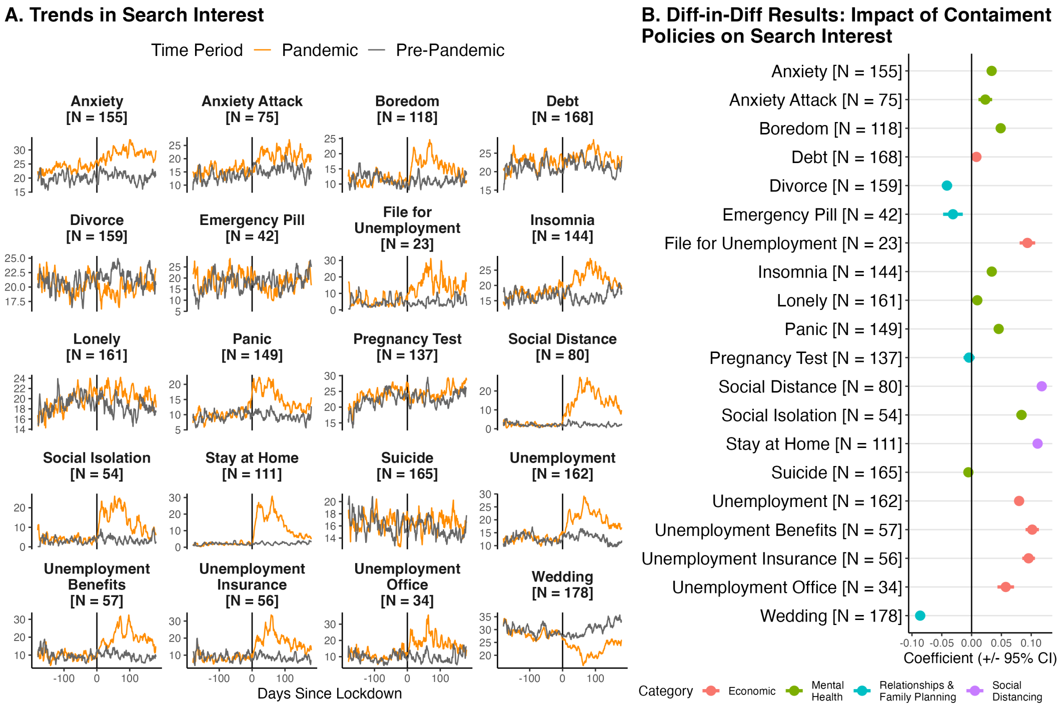

Figure S25: Association of COVID-19 policies with search interest: results pooling all countries. Point estimates and 95% confidence intervals are shown. To more clearly show trends, the seven day moving average of search interest is shown in panel A. 'N' indicates the number of countries with available data.

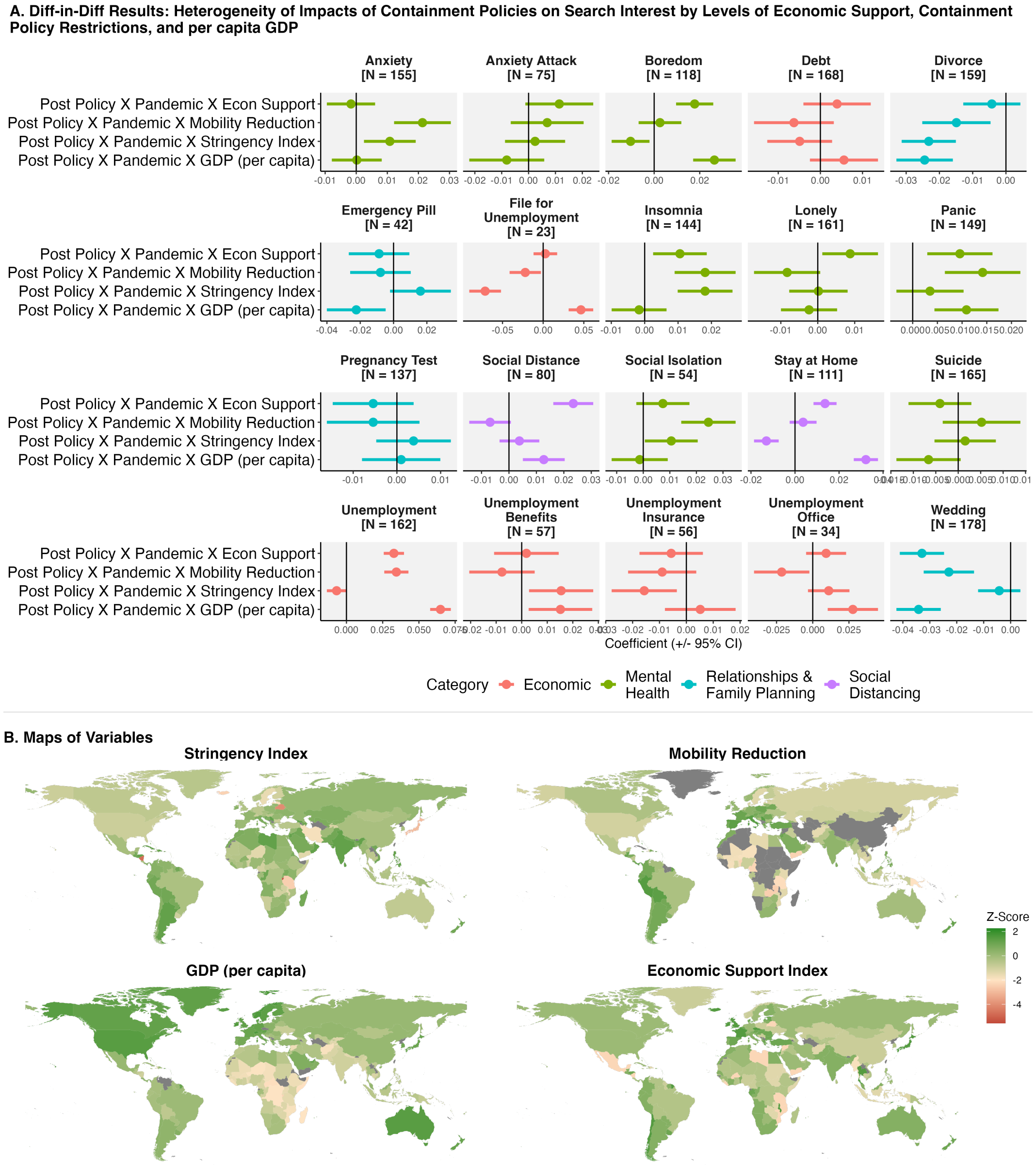

Figure S26: Association of COVID-19 policies with search interest: difference-in-differences results that explore heterogeneity of results across containment policy restrictiveness, economic support, and GDP per capita. Each coefficient comes from a separate regression. The stringency index comes from the University of Oxford COVID-19 Government Response tracker, a composite measure of the restrictiveness of policy measures. Mobility reduction comes from Google COVID-19 Community Mobility Reports, which measure the percent change in mobility relative to pre-pandemic levels. Per capita GDP comes from the World Bank's World Development Indicators; we use log per capita GDP. The Economic Support index from the Oxford COVID-19 Government Response tracker, which measures the extent of economic support across metrics such as income support and debt relief. We standardize all variables into z-scores—having a mean of zero and standard deviation of one. 'N' indicates the number of countries with available data. Maps produced using R, version 4.2.2 (`https://www.r-project.org/`); data for country boundaries come from Natural Earth (`https://www.naturalearthdata.com/`).

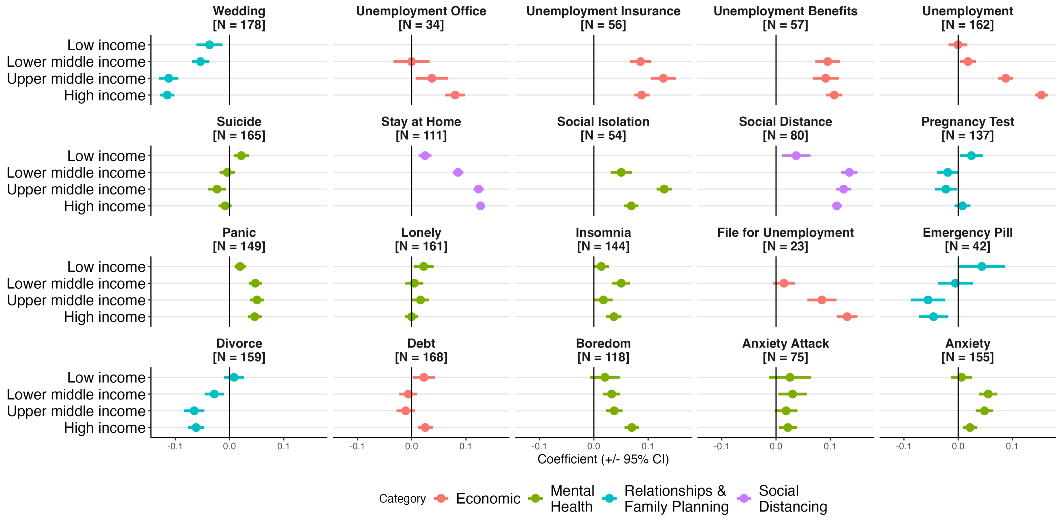

Figure S27: Association of COVID-19 policies with search interest: difference-in-difference results pooling countries by income level. Point estimates and 95% confidence intervals are shown. 'N' indicates the number of countries with available data.

References

[1] Thomas Beaney, Jonathan M Clarke, Vageesh Jain, Amelia Kataria Golestaneh, Gemma Lyons, David Salman, and Azeem Majeed. Excess mortality: the gold standard in measuring the impact of covid-19 worldwide? *Journal of the Royal Society of Medicine*, 113(9):329–334, 2020. PMID: 32910871.

[2] Google Help. FAQ about google trends data. `https://support.google.com/trends/answer/4365533?hl=en`. Accessed: 2022-01-15.

[3] Stephan Kolassa (https://stats.stackexchange.com/users/1352/stephan kolassa). scale a number between a range. Cross Validated. URL:https://stats.stackexchange.com/q/281164 (version: 2018-04-19).

[4] World Health Organization. Global excess deaths associated with covid-19: Modelled estimates, 2022. Accessed on: 2023-01-21.
